# Vitamin D and risk of pregnancy related hypertensive disorders: mendelian randomisation study

**DOI:** 10.1136/bmj.k2167

**Published:** 2018-06-21

**Authors:** Maria C Magnus, Kozeta Miliku, Anna Bauer, Stephanie M Engel, Janine F Felix, Vincent W V Jaddoe, Debbie A Lawlor, Stephanie J London, Per Magnus, Ralph McGinnis, Wenche Nystad, Christian M Page, Fernando Rivadeneira, Lars C Stene, German Tapia, Nicholas Williams, Carolina Bonilla, Abigail Fraser

**Affiliations:** 1 Medical Research Council Integrative Epidemiology Unit, University of Bristol, Bristol BS8 2BN, UK; 2 Department of Population Health Sciences, Bristol Medical School, Bristol, UK; 3 Centre for Fertility and Health, Norwegian Institute of Public Health, Oslo, Norway; 4 Generation R Study Group, Erasmus MC, University Medical Centre Rotterdam, Rotterdam, Netherlands; 5 Department of Pediatrics, Erasmus MC, University Medical Centre Rotterdam, Rotterdam, Netherlands; 6 Department of Epidemiology, Erasmus MC, University Medical Centre Rotterdam, Rotterdam, Netherlands; 7 Gillings School of Global Public Health, University of North Carolina, Chapel Hill, NC, USA; 8 National Institute for Health Research Bristol Biomedical Research Centre, University Hospitals Bristol NHS Foundation Trust, and the University of Bristol, Bristol, UK; 9 Epidemiology Branch, National Institute of Environmental Health Sciences, National Institutes of Health, Department of Health and Human Services, Research Triangle Park, NC, USA; 10 Sanger Institute, University of Cambridge, Cambridge, UK; 11 Division for Mental and Physical Health, Norwegian Institute of Public Health, Oslo, Norway; 12 Oslo Centre for Biostatistics and Epidemiology, Oslo University Hospital, Oslo, Norway; 13 Department of Internal Medicine, Erasmus MC, University Medical Centre Rotterdam, Rotterdam, Netherlands; 14 Departamento de Medicina Preventiva, Faculdade de Medicina, Universidade de São Paulo, São Paulo, Brazil

## Abstract

**Objective:**

To use mendelian randomisation to investigate whether 25-hydroxyvitamin D concentration has a causal effect on gestational hypertension or pre-eclampsia.

**Design:**

One and two sample mendelian randomisation analyses.

**Setting:**

Two European pregnancy cohorts (Avon Longitudinal Study of Parents and Children, and Generation R Study), and two case-control studies (subgroup nested within the Norwegian Mother and Child Cohort Study, and the UK Genetics of Pre-eclampsia Study).

**Participants:**

7389 women in a one sample mendelian randomisation analysis (751 with gestational hypertension and 135 with pre-eclampsia), and 3388 pre-eclampsia cases and 6059 controls in a two sample mendelian randomisation analysis.

**Exposures:**

Single nucleotide polymorphisms in genes associated with vitamin D synthesis (rs10741657 and rs12785878) and metabolism (rs6013897 and rs2282679) were used as instrumental variables.

**Main outcome measures:**

Gestational hypertension and pre-eclampsia defined according to the International Society for the Study of Hypertension in Pregnancy.

**Results:**

In the conventional multivariable analysis, the relative risk for pre-eclampsia was 1.03 (95% confidence interval 1.00 to 1.07) per 10% decrease in 25-hydroxyvitamin D level, and 2.04 (1.02 to 4.07) for 25-hydroxyvitamin D levels <25 nmol/L compared with ≥75 nmol/L. No association was found for gestational hypertension. The one sample mendelian randomisation analysis using the total genetic risk score as an instrument did not provide strong evidence of a linear effect of 25-hydroxyvitamin D on the risk of gestational hypertension or pre-eclampsia: odds ratio 0.90 (95% confidence interval 0.78 to 1.03) and 1.19 (0.92 to 1.52) per 10% decrease, respectively. The two sample mendelian randomisation estimate gave an odds ratio for pre-eclampsia of 0.98 (0.89 to 1.07) per 10% decrease in 25-hydroxyvitamin D level, an odds ratio of 0.96 (0.80 to 1.15) per unit increase in the log(odds) of 25-hydroxyvitamin D level <75 nmol/L, and an odds ratio of 0.93 (0.73 to 1.19) per unit increase in the log(odds) of 25-hydroxyvitamin D levels <50 nmol/L.

**Conclusions:**

No strong evidence was found to support a causal effect of vitamin D status on gestational hypertension or pre-eclampsia. Future mendelian randomisation studies with a larger number of women with pre-eclampsia or more genetic instruments that would increase the proportion of 25-hydroxyvitamin D levels explained by the instrument are needed.

## Introduction

Low circulating 25-hydroxyvitamin D levels are common in pregnant women.^1^ The biologically active form of vitamin D, 1,25-dihydroxyvitamin D3 (calcitriol), can suppress renin biosynthesis and vascular smooth muscle cell proliferation, modulates macrophage activity and cytokine production,^2^ ^3^ and regulates transcription of genes linked to placental invasion, normal implantation, and angiogenesis.^4^ Therefore, vitamin D status is a plausible causal factor in gestational hypertension and pre-eclampsia.

Meta-analyses of observational studies suggest an inverse association between 25-hydroxyvitamin D concentration and pre-eclampsia, but the potential for bias remains.^5^ ^6^ A recent meta-analysis of three high quality randomised controlled trials that evaluated vitamin D supplementation during pregnancy found no strong evidence of a protective effect on gestational hypertension (pooled risk ratio 1.69, 95% confidence interval 0.73 to 3.92) or pre-eclampsia (1.09, 0.43 to 2.76).^7^

Triangulating findings from studies that use different designs and analytical approaches increases confidence in findings.^8^ Mendelian randomisation analysis uses genetic variants as instrumental variables to examine whether an association is causal because the allocation of genes at conception is random at the population level and therefore independent of confounding factors.^9^ ^10^ This approach examines the association of genetically determined levels of the exposure in relation to the outcomes of interest. A one sample mendelian randomisation analysis requires information on the genetic variants, exposure, and outcome from the same individual. A two sample mendelian randomisation analysis uses estimates of the association between the genetic variants and the exposure from one sample and the association between the genetic variants and the outcome from a second sample. One sample mendelian randomisation provides researchers with greater control of the analysis—for example, they can determine how to analyse the exposure (as a continuum, or in categories) and examine the association between the genetic instruments and measured confounders. Two sample mendelian randomisation takes advantage of published summary estimates from large scale genome wide association studies, which often results in greater statistical power. Genome wide association studies show robust associations of genetic variants located in genes that act in the pathway for vitamin D synthesis (*CYP2R1* and *DHCR7*/*NADSYN1*) and metabolism (eg, *CYP24A1* and *GC*) with 25-hydroxyvitamin D level.^11^ ^12^ If the alleles associated with lower 25-hydroxyvitamin D concentrations are also associated with a greater risk of gestational hypertension or pre-eclampsia, this points towards a causal effect of lower 25-hydroxyvitamin D levels.

We investigated the causal effect of 25-hydroxyvitamin D level on pregnancy related hypertensive disorders using genetic variants that are associated with 25-hydroxyvitamin D levels as instrumental variables in a mendelian randomisation analysis.

## Methods

### Study population for multivariable regression and one sample mendelian randomisation analyses

Information on genetic variants, antenatal 25-hydroxyvitamin D levels, gestational hypertension, and pre-eclampsia were available in the Avon Longitudinal Study of Parents and Children (ALSPAC)^13^ ^14^ and the Generation R Study^15^ (see the online supplementary material for more details). Written informed consent was obtained from all participants. We excluded women with multiple births, of non-European ethnicity, and with hypertension before pregnancy. Women of non-European ethnicity included those who were not of European ancestry based on self reported information or principal component analysis of their genome. A total of 4066 women from ALSPAC and 3323 women from Generation R were available for analysis (eFigs 1 and 2).

### Measurement of antenatal 25-hydroxyvitamin D levels

Antenatal 25-hydroxyvitamin D levels were measured in serum (ALSPAC) or plasma (Generation R) using liquid chromatography-tandem mass spectrometry. 25-hydroxyvitamin D levels were natural log transformed and standardised according to calendar time of blood sampling by adding to the population mean each person’s residual from a linear regression model, including the sine cosine function of calendar time of blood sampling as predictors. Additional information about the adjustment for season is available in the online supplementary material. For the conventional multivariable analysis, we evaluated 25-hydroxyvitamin D levels continuously (by 10% decrease) and also categorised according to level based on recommendations of the Endocrine Society: less than 25 nmol/L, 25-49.9 nmol/L, 50-74.9 nmol/L, and 75 nmol/L or higher.^16^ We did not have adequate power to test non-linear associations in the one sample mendelian randomisation analysis as there were only 751 women with gestational hypertension (592 in ALSPAC and 159 in Generation R) and 135 women with pre-eclampsia (77 in ALSPAC and 58 in Generation R) across the two cohorts.

### Study population for the two sample mendelian randomisation analysis

We also conducted a two sample mendelian randomisation analysis of pre-eclampsia using estimates of the associations between the genetic instruments and 25-hydroxyvitamin D l levels, and the associations between the genetic instruments and pre-eclampsia from two independent samples. We used published estimates of the associations between the genetic instruments and 25-hydroxyvitamin D levels from 21 European cohorts (n=42 024).^17^ Estimates of the association between the genetic variants and pre-eclampsia were available from two case-control studies: a subgroup of individuals from a validation study of pre-eclampsia nested within the Norwegian Mother and Child Cohort Study (MoBa)^18^ (1513 pre-eclampsia cases and 971 healthy controls) and the UK Genetics of Pre-eclampsia study (GOPEC)^19^ (1875 pre-eclampsia cases and 5088 healthy controls). Additional information is available in the online supplementary material.

### Genetic instruments for the one and two sample mendelian randomisation analyses

Our theoretical framework is shown in eFig 3. Genetic instruments were four single nucleotide polymorphisms (SNPs) in genes associated with vitamin D synthesis (*CYP2R1*/rs10741657 and *DHCR7*/rs12785878) and metabolism (*CYP24A1*/rs6013897 and *GC*/rs2282679), which have been confirmed to be associated with 25-hydroxyvitamin D levels.^11^ ^12^ Information about the genotyping and quality control procedures is available in the online supplementary material. The genetic instruments were considered individually and combined into synthesis, metabolism, and total genetic risk scores. We created weighted genetic risk scores by weighting the instruments by the published effect estimates that we used for our two sample mendelian randomisation.^17^ We also did two separate two sample mendelian randomisation analyses of 25-hydroxyvitamin D levels less than 75 nmol/L (compared with 75 nmol/L or more) and less than 50 nmol/L (compared with 50 nmol/L or more) for pre-eclampsia; we used published estimates of the association between three instruments (rs10741657, rs12785878, and rs2282679) with these 25-hydroxyvitamin D thresholds.^12^ Associations between the genetic instruments and low levels of 25-hydroxyvitamin D —such as less than 25 nmol/L—have not been published. The four SNPs were in Hardy-Weinberg equilibrium (P≥0.01) and none was in linkage disequilibrium (R^2^≤0.01).

### Definition of gestational hypertension and pre-eclampsia

All cohorts assessed pre-existing hypertension by self report. Routine blood pressure and proteinuria measurements from antenatal medical records were used to define gestational hypertension and pre-eclampsia. Gestational hypertension and pre-eclampsia were defined according to the criteria of the International Society for the Study of Hypertension in Pregnancy.^20^ Women without pre-existing hypertension were classified as having gestational hypertension if they had a systolic blood pressure ≥140 mm Hg and/or diastolic blood pressure ≥90 mm Hg on at least two occasions first occurring after 20 gestational weeks. Pre-eclampsia was defined as gestational hypertension in combination with proteinuria (≥0.3 g/d). For the multivariable analysis and the one sample mendelian randomisation analysis, the outcome was categorised as women without any hypertension either before or during pregnancy (reference group), gestational hypertension only, and pre-eclampsia.

### Covariates adjusted for in multivariable regression analysis

Information on covariates was available for the multivariable analysis: age, parity (0, 1, 2, and ≥3 births), prepregnancy body mass index (<18.5, 18.5-24.9, 25-29.9, and ≥30 kg/m^2^), educational level (low, medium, and high), smoking status during pregnancy (never, former/until pregnancy was known, and continued smoking), serum calcium concentration (nmol/L) (ALSPAC)/energy adjusted calcium intake (mg) (Generation R), and gestational week of sample collection. We also extracted information on any intake of vitamin D supplements during pregnancy, defined as either a pure vitamin D supplement, a multivitamin, or cod liver oil.

### Statistical analysis

We ran all analyses separately for each cohort and combined the results of each cohort using a random effects meta-analysis. The degree of heterogeneity between the cohorts was estimated using the I^2^ statistic.

We first estimated the multivariable adjusted associations of 25-hydroxyvitamin D level with gestational hypertension and pre-eclampsia using multinomial logistic regression in ALSPAC and Generation R. Coefficients were multiplied by ln(0.9) to reflect the association per 10% decrease in 25-hydroxyvitamin D level.^21^ Missing data on covariates ranged from 0% to 13% in the two cohorts (eTable 1). Multivariable imputation of missing covariate values was done with chained equations (ALSPAC), or according to the fully conditional specification method predictive mean matching (Generation R). Twenty datasets with imputed values were generated. Details of how these methods were applied and comparison of observed and imputed data are given in the online supplementary material and eTable 2, respectively.

We estimated the association between the genetic instruments and natural log transformed 25-hydroxyvitamin D values using linear regression. The exponentiated coefficients were multiplied by 100 (exp(coef)−1×100) to reflect the per cent change in 25-hydroxyvitamin D level per increase in copy number of the risk allele associated with lower 25-hydroxyvitamin D levels,^21^ and we report the R^2^ and F statistic as indicators of the strength of the instruments. Our assumption in the mendelian randomisation analyses is that genetic variants will not be associated with confounding factors, and we tested this for observed confounders.

The associations of the genetic instruments with gestational hypertension and pre-eclampsia were estimated by multinomial logistic regression analysis, adjusting for seven genomic principal components in ALSPAC. Generation R did not have information on genome wide genotypes to generate principal components. We also estimated the magnitude of the causal effect of 25-hydroxyvitamin D level on gestational hypertension and pre-eclampsia using a two step instrumental variable analysis. Firstly, we estimated the genetically predicted 25-hydroxyvitamin D values from a linear regression, where log(25-hydroxyvitamin D) was the outcome and the genetic instrument the exposure. Secondly, we used these genetically predicted 25-hydroxyvitamin D levels as the exposure in a logistic regression model of the outcomes of interest, adjusting for seven principal components (ALSPAC only). The standard error of the coefficients from the logistic regression model were estimated using bootstrapping. We then compared the estimates obtained from the multivariable regression analysis with the estimates from the instrumental variable analysis using a non-parametric bootstrapping test. The multivariable and one sample mendelian randomisation analyses were done in Stata version 14.

We obtained estimates of the association between the genetic variants and pre-eclampsia for the two sample mendelian randomisation analysis using logistic regression, adjusting for the first five principal components. We then estimated the causal effect of 25-hydroxyvitamin D level on pre-eclampsia with each SNP as an instrument using the Wald ratio. We combined the estimates of the causal effects of each single SNP as an instrument using an inverse variance weighted method, which is a linear regression analysis through the mean 25-hydroxyvitamin D level and proportion with pre-eclampsia for each individual SNP.^22^ This method assumes that there are no alternative ways for the genetic instruments to influence the risk of gestational hypertension or pre-eclampsia other than through 25-hydroxyvitamin D (no horizontal pleiotropy). To assess this assumption, we estimated the causal effect using mendelian randomisation-Egger regression, which is a similar regression method that does not force the regression line through the intercept.^23^ A non-zero intercept value from mendelian randomisation-Egger regression is an indicator of possible horizontal pleiotropy, whereas the slope provides an unbiased estimate in the presence of pleiotropy with the assumption that the pleiotropic effects of the genetic instruments are uncorrelated with their associations with the exposure.^23^ We assessed heterogeneity in the estimated causal effect using the individual genetic instruments with the Q statistic. For the secondary analysis of the two 25-hydroxyvitamin D cut-off levels, the associations are the odds ratio for pre-eclampsia per unit increase in the log(odds) of having a 25-hydroxyvitamin D level below the cut-off. The two sample mendelian randomisation analysis was done in R 3.2.2 (R Foundation, www.R-project.org).

### Patient and public involvement

No patients were involved in setting the research question or the outcome measures, nor were they involved in developing plans for recruitment, design, or implementation of the study. No patients were asked to advise on interpretation or writing up of results. There are no plans to disseminate the results of the research to study participants or the relevant patient community.

## Results

### Distribution of 25-hydroxyvitamin D and genetic instruments by background characteristics

Supplementary eTable 1 shows the characteristics of the 7389 women included in the one sample mendelian randomisation analysis. In the two cohorts, 751 women had gestational hypertension (592 in ALSPAC and 159 in Generation R) and 135 had pre-eclampsia (77 in ALSPAC and 58 in Generation R). The distribution of background characteristics was similar in the observed and imputed datasets (eTable 2). 25-hydroxyvitamin D level was positively associated with age (difference 1-2 years) and education (difference in proportion with high education 7-9%) in both cohorts, and inversely associated with smoking (difference in proportion of women who smoked during pregnancy 13-20%) and body mass index (difference in proportion with normal body mass index 6-38%) (eTables 3 and 4). In contrast, the genetic instruments showed no strong evidence of associations with the confounding factors (eTables 5 and 6). Notably, we did not observe strong evidence of an association between intake of vitamin D supplements and the genetic instruments.

### Association of 25-hydroxyvitamin D levels with gestational hypertension and pre-eclampsia: multivariable regression analysis

We did not find strong evidence of any (linear or non-linear) association between 25-hydroxyvitamin D level and gestational hypertension in the multivariable analysis (eTable 7). However, we observed weak evidence of a linear association between 25-hydroxyvitamin D level and pre-eclampsia (pooled adjusted relative risk 1.03 (95% confidence interval 1.00 to 1.07) per 10% decrease; eTable 7). A 25-hydroxyvitamin D level less than 25 nmol/L was associated with a twofold increased risk of pre-eclampsia compared with levels of 75 nmol/L or more (pooled adjusted relative risk 2.04 (1.02 to 4.07); eTable 7).

### Strength of genetic variants as instruments for 25-hydroxyvitamin D level

Supplementary eTable 8 shows the strength of the genetic instruments in predicting 25-hydroxyvitamin D level in ALSPAC and Generation R. The synthesis score explained 0.2% (F=9, n=4062 and 0.6% (F=21, n=3275) of the variation in 25-hydroxyvitamin D levels in ALSPAC and Generation R, respectively. In comparison, the metabolism score explained 1.2% (F=49, n=3920) and 1.5% (F=52, n=3284) of the variation in 25-hydroxyvitamin D levels in ALSPAC and Generation R, respectively. For the published estimates of the associations of the genetic instruments and 25-hydroxyvitamin D level used for the two sample mendelian randomisation analysis, the synthesis score explained 0.6% of the variation in 25-hydroxyvitamin D levels (F=230, n=35 873), whereas the metabolism score explained 1.3% of the variation in 25-hydroxyvitamin D levels (F=489, n=38 191).^17^

### Association of 25-hydroxyvitamin D level with gestational hypertension and pre-eclampsia: one sample mendelian randomisation analysis

**Fig 1:**
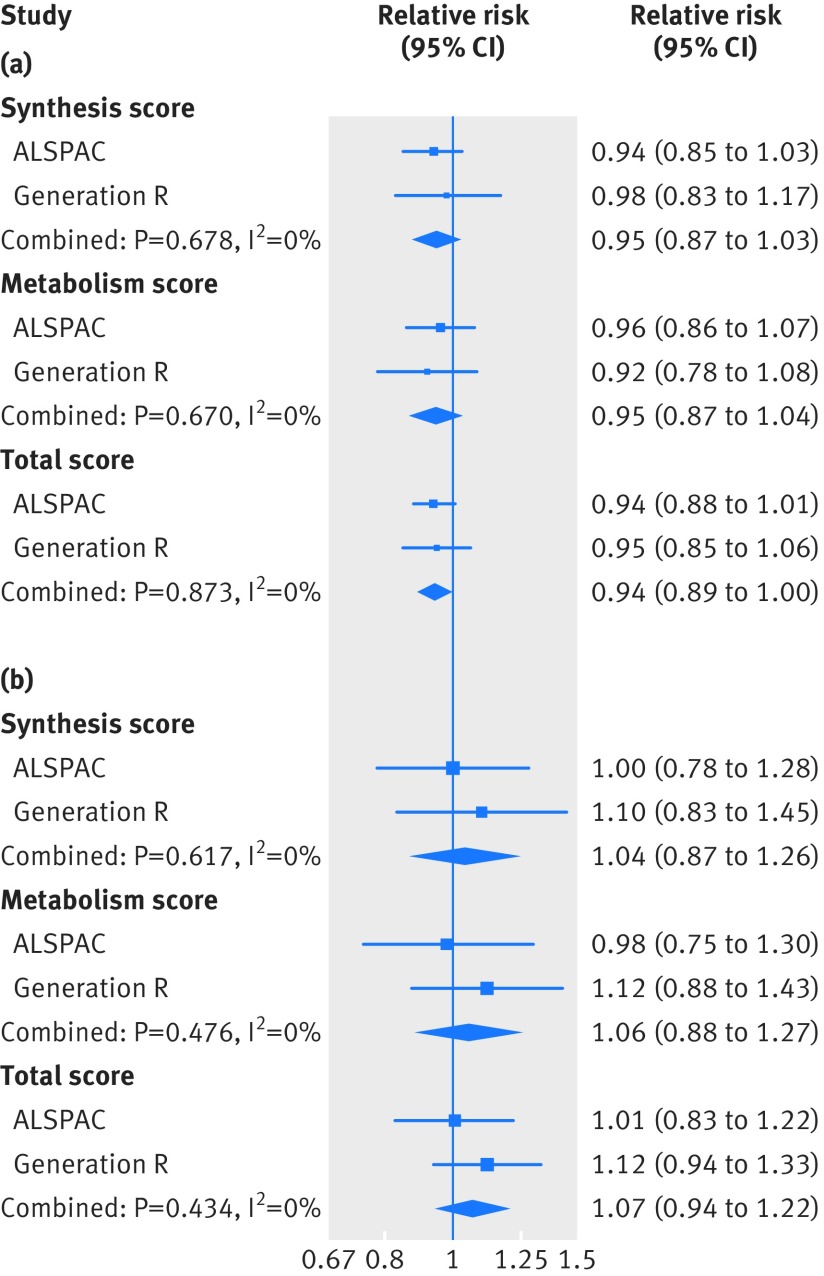
Associations of the three genetic risk scores for 25-hydroxyvitamin D level with gestational hypertension and pre-eclampsia from the one sample mendelian randomisation analysis of the Avon Longitudinal Study of Parents and Children (ALSPAC) and the Generation R Study. (a) Gestational hypertension, (b) pre-eclampsia. Measures of association were obtained from multinomial logistic regression analysis. Associations reflect the additive risk of each additional copy of the risk allele associated with decreased 25-hydroxyvitamin D levels, and are adjusted for seven principal components to account for population stratification (ALSPAC only)

**Fig 2:**
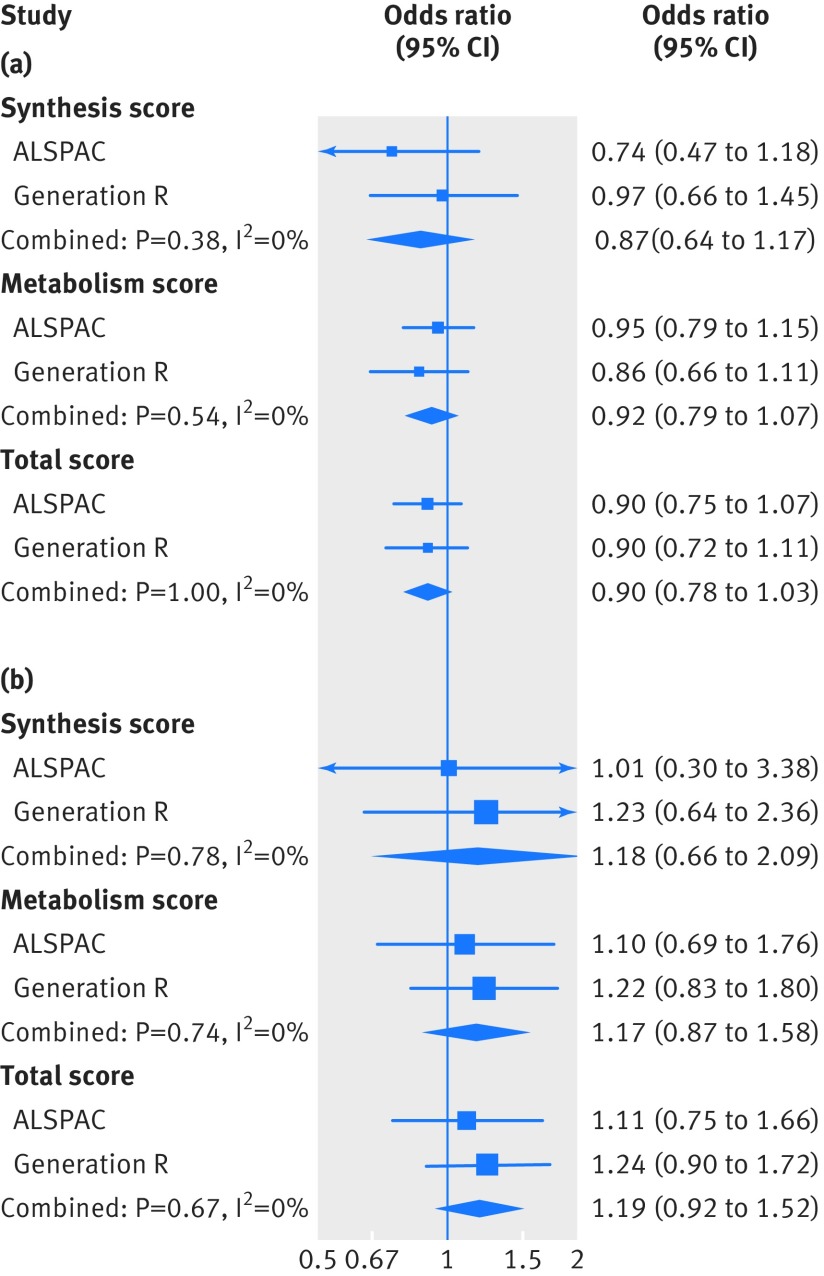
Causal associations of 25-hydroxyvitamin D level with gestational hypertension and pre-eclampsia from the one sample mendelian randomisation analysis of the Avon Longitudinal Study of Parents and Children (ALSPAC) and the Generation R Study. (a) Gestational hypertension, (b) pre-eclampsia. The causal association was estimated using two step instrumental variable analysis, and associations reflect the change in risk per 10% decrease in 25-hydroxyvitamin D levels. The associations are adjusted for gestational week of blood sampling and seven principal components to account for population stratification (ALSPAC only)

We found no consistent evidence of any associations of the three genetic risk scores with gestational hypertension or pre-eclampsia in the two cohorts (fig 1). When we evaluated the four SNPs individually, we found weak evidence of an association between a greater copy number of the 25-hydroxyvitamin D risk allele in rs2282679 and pre-eclampsia (eTable 9). The instrumental variable analysis also showed no consistent evidence of a causal linear effect of 25-hydroxyvitamin D level on the risk of gestational hypertension or pre-eclampsia: the odds ratio for gestational hypertension was 0.90 (95% confidence interval 0.78 to 1.03) per 10% decrease in 25-hydroxyvitamin D level, and for pre-eclampsia was 1.19 (0.92 to 1.52) per 10% decrease in 25-hydroxyvitamin D level, when using the total genetic risk score as the instrument (fig 2 and eTable 10). When we compared the results from multivariable and instrumental variable analyses, no strong evidence was found that the estimates differed (in ALSPAC, P=0.17 for gestational hypertension and 0.77 for pre-eclampsia; in Generation R, P=0.10 for gestational hypertension and 0.12 for pre-eclampsia). Based on a question raised in the review process, since rs6013897 was not a strong instrument for 25-hydroxyvitamin D levels, we conducted an analysis where we excluded rs6013897 from the total genetic risk score. This sensitivity analysis did not change our findings: the odds ratio for gestational hypertension was 0.91 (0.80 to 1.04) per 10% decrease in 25-hydroxyvitamin D level, and for pre-eclampsia was 1.21 (0.94 to 1.57) per 10% decrease in 25-hydroxyvitamin D level.

### Association of 25-hydroxyvitamin D level with pre-eclampsia: two sample mendelian randomisation analysis

**Fig 3:**
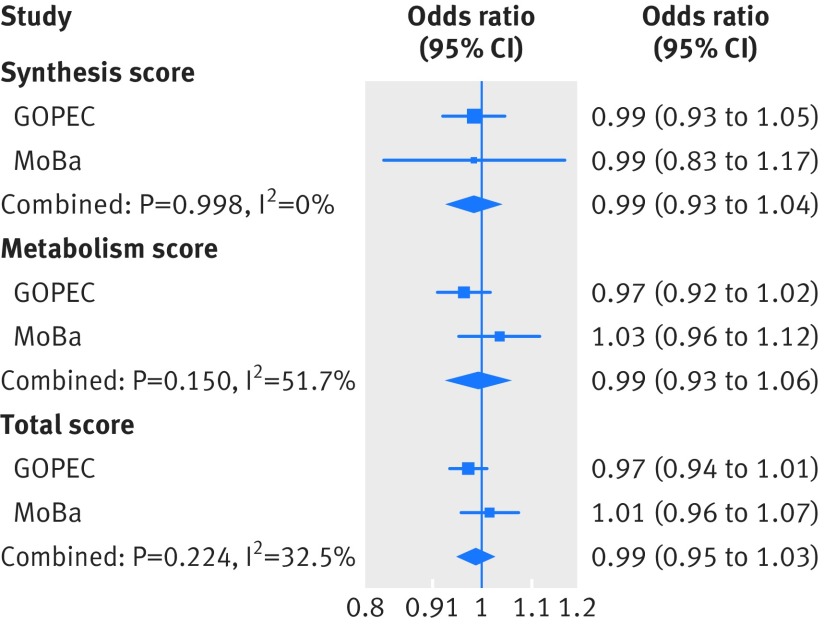
Associations of the three genetic risk scores for 25-hydroxyvitamin D level with pre-eclampsia from the two sample mendelian randomisation analysis of the UK Genetics of Pre-eclampsia Study (GOPEC) and the Norwegian Mother and Child Cohort Study (MoBa). The associations were estimated by ordinary logistic regression analysis, and reflect the additive risk of each additional copy of the risk allele associated with decreased 25-hydroxyvitamin D levels. The estimates are adjusted for five principal components

**Fig 4:**
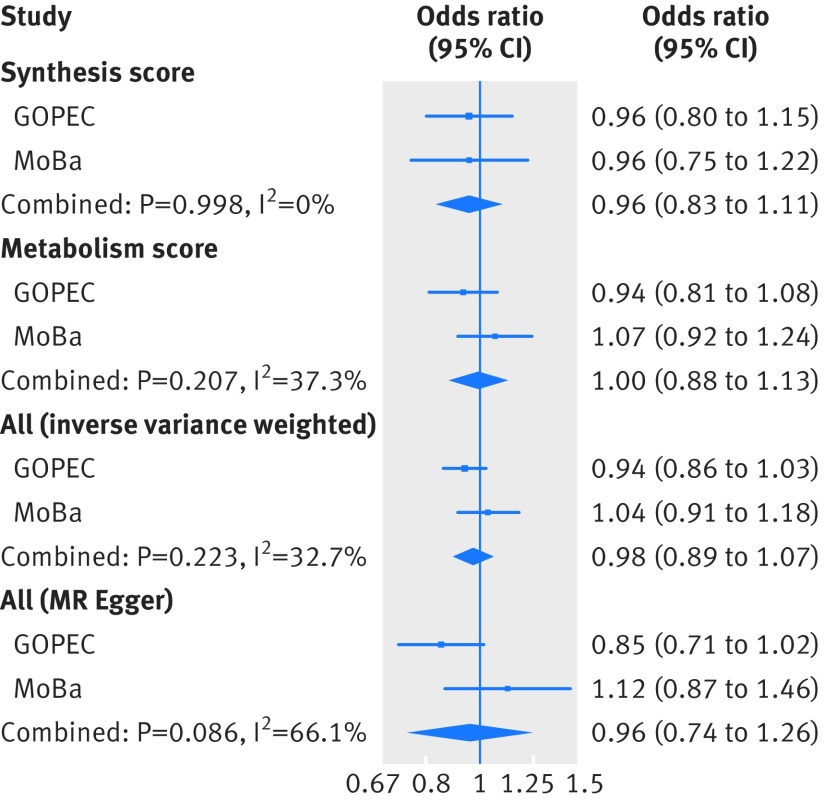
Causal association between 25-hydroxyvitamin D level and pre-eclampsia from the two sample mendelian randomisation (MR) analysis of the UK Genetics of Pre-eclampsia Study (GOPEC) and the Norwegian Mother and Child Cohort Study (MoBa). Associations are estimated using the inverse variance weighted and MR-Egger methods, and reflect the change in risk per 10% decrease in 25-hydroxyvitamin D levels

**Fig 5:**
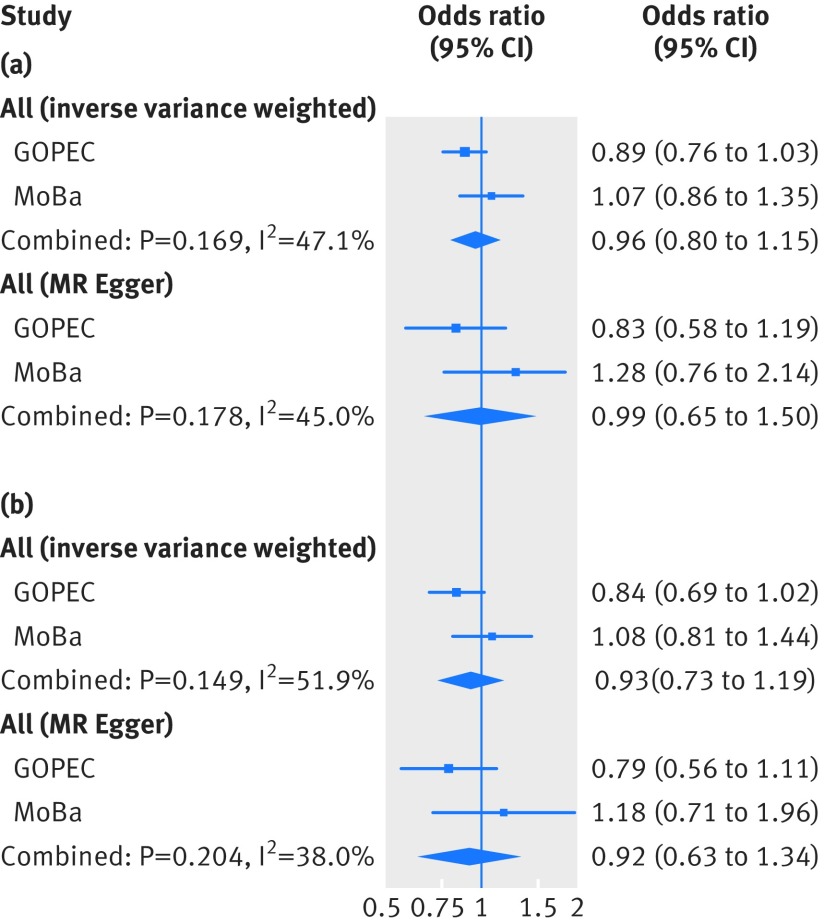
Causal association between 25-hydroxyvitamin D cut-off levels and pre-eclampsia from the two sample mendelian randomisation (MR) analysis of the UK Genetics of Pre-eclampsia Study (GOPEC) and the Norwegian Mother and Child Cohort Study (MoBa). (a) <75 nmol/L compared with ≥75 nmol/L, (b) <50 nmol/L compared with ≥50 nmol/L. Associations reflect the risk of pre-eclampsia per increase in the log(odds) of the risk of being below the respective cut-off level for 25-hydroxyvitamin D

Supplementary eTable11 shows the risk allele frequencies in the two-case control samples of pre-eclampsia (MoBa and GOPEC). Similar to the one sample mendelian randomisation analysis, we found no associations between the genetic instruments for 25-hydroxyvitamin D levels and pre-eclampsia in the two sample mendelian randomisation analysis (fig 3 and eTable 12), and no evidence of a causal linear effect in the formal two sample mendelian randomisation analysis, (odds ratio 0.98 (95% confidence interval 0.89 to 1.07) per 10% decrease in 25-hydroxyvitamin D level (fig 4 and eTable 13). The P values from the Q statistic were 0.58 in GOPEC and 0.89 in MoBa (inverse variance weighted method), indicating no evidence of heterogeneity in the estimates of the genetic instruments. Furthermore, we found no strong evidence of horizontal pleiotropy: mendelian randomisation-Egger intercept −0.06 (P=0.34) in GOPEC and 0.04 (P=0.56) in MoBa. Similar to what we observed in the one sample mendelian randomisation analysis, excluding rs6013897 from the total genetic risk score did not change our findings, odds ratio 0.97 (0.88 to 1.08) per 10% decrease in 25-hydroxyvitamin D levels. In the two sample mendelian randomisation analysis of 25-hydroxyvitamin D cut-off levels, no strong evidence was found of a causal effect of 25-hydroxyvitamin D level on the risk of pre-eclampsia: the odds ratio for pre-eclampsia was 0.96 (0.80 to 1.15) per unit increase in the log(odds) of having a 25-hydroxyvitamin D level less than 75 nmol/L and 0.93 (0.73 to 1.19) per unit increase in the log(odds) of having a 25-hydroxyvitamin D level less than 50 nmol/L (fig 5 and eTable 14).

## Discussion

A lower 25-hydroxyvitamin D level was weakly associated with a lower risk of gestational hypertension and higher risk of pre-eclampsia in the one sample mendelian randomisation analysis. This could indicate that the null association between 25-hydroxyvitamin D level and gestational hypertension in the multivariable analysis was influenced by unobserved confounding. Further evidence from larger studies is needed to provide conclusive evidence. Furthermore, no evidence was found of an association between 25-hydroxyvitamin D level and pre-eclampsia in the larger two sample mendelian randomisation. Nor did we find any appreciable evidence of a causal effect of 25-hydroxyvitamin D levels less than 75 nmol/L or less than 50 nmol/L on the risk of pre-eclampsia.

### Strengths and limitations of this study

The main strengths of our study are the use of genetic variants as instrumental variables to reduce the possibility of confounding, and our inclusion of multiple cohorts. The methods for ascertaining outcome were similar for the cohorts but we acknowledge a difference in the proportion of women with gestational hypertension in ALSPAC (15%) and Generation R (5%) cohorts. This is likely explained by the fact that Generation R relied on registered diagnoses in the antenatal charts, whereas ALSPAC abstracted blood pressure and proteinuria measures from the charts and then defined the outcomes according to the guidelines. We accounted for some observed heterogeneity in the associations between the cohorts, which might be explained by the difference in ascertaining the outcome, by running a random effects meta-analysis instead of a fixed effects meta-analysis. Mendelian randomisation analysis has limitations. Horizontal pleiotropy could bias our findings. We observed no consistent evidence of associations between the genetic instruments and a range of background characteristics in the two pregnancy cohorts included in the one sample mendelian randomisation analysis. This might be explained by the modest sample size. However, a study of 6877 Europeans in the 1958 British Birth Cohort reported no associations between the four genetic instruments that we used in the current study and a wide range of potential pleiotropic pathways, including oily fish consumption, smoking, body mass index, abdominal obesity, social class, and C reactive protein levels.^24^ Therefore, no strong evidence exists of pleiotropy for the genetic instruments for 25-hydroxyvitamin D. This is further supported by the findings of the mendelian randomisation-Egger regression that had an intercept of −0.06 (P=0.34) in GOPEC and 0.04 (P=0.56) in MoBa.^23^ Our analyses were restricted to pregnant women. If vitamin D status affects fertility, this might have resulted in selection bias.^25^ ^26^

We explored the association between the genetic instruments and intake of vitamin D supplements because if women with lower genetically predicted 25-hydroxyvitamin D levels are more likely to take supplements, this could theoretically distort our findings. The proportion of women who took vitamin D supplements during pregnancy differed in the two cohorts, which might reflect cultural, socioeconomic, or policy differences. However, we did not see strong evidence of an association between the genetic instruments and intake of vitamin D supplements in ALSPAC or Generation R. In addition, associations between the genetic instruments and measured 25-hydroxyvitamin D levels were similar to previous reports.

Finally, in the two sample mendelian randomisation analysis, we used estimates for the associations of the genetic instruments with 25-hydroxyvitamin D levels and pre-eclampsia from the largest samples available. Our two sample mendelian randomisation analysis of pre-eclampsia (3388 cases and 6059 controls), using the total genetic risk score as an instrument (explaining about 2% of the variation in 25-hydroxyvitamin D levels), was adequately powered (80%) to detect an odds ratio of 1.5 per standard deviation decrease in 25-hydroxyvitamin D level.^27^ Translating this to the association we were able to detect for a 10% decrease in 25-hydroxyvitamin D level (0.105 on the log scale), using the estimate of the standard deviation of natural log transformed 25-hydroxyvitamin D level in ALSPAC (0.45), this is equivalent to an odds of 1.10 per 10% decrease in 25-hydroxyvitamin D level (exp ((ln(1.5)/0.45)×0.105). We therefore acknowledge that a more modest effect might be present that we were not able to detect. Future mendelian randomisation studies with a larger number of women with pre-eclampsia are therefore needed.

### Comparison with other studies

Previous observational studies of 25-hydroxyvitamin D level and risk of pre-eclampsia report conflicting findings, as summarised in meta-analyses,^5^ ^6^ which report a pooled odds ratio for pre-eclampsia of 1.70 (95% confidence interval 1.25 to 2.58) when comparing women with 25-hydroxyvitamin D levels less than 75 nmol/L with those with levels of 75 nmol/L or more.^5^ Estimates of the associations vary according to study design, whether the original studies had adjusted for important confounders, the categorisation of 25-hydroxyvitamin D, and 25-hydroxyvitamin D quantification method. Previous studies of 25-hydroxyvitamin D level and gestational hypertension also reported conflicting findings.^28^ ^29^ ^30^ ^31^

Our findings are more in line with a recent systematic review and meta-analysis of randomised controlled trials of vitamin D supplementation in pregnancy. The pooled risk ratio of pre-eclampsia was 1.09 (95% confidence interval 0.43 to 2.76), with a high degree of heterogeneity in the effects estimates between the three included trials (I^2^=67%).^7^ The sizes of the trials were modest, ranging between about 70 and 200 women in each study arm.^32^ ^33^ ^34^ The intervention also varied between the three studies, with 50 000 IU (1250 µg) of vitamin D3 given once every two weeks in two trials and 4000 (100 µg) IU vitamin D3 given daily in one trial.^32^ ^33^ ^34^ The authors of the meta-analysis attribute the difference between their pooled estimate and the results of previous meta-analyses to their stricter exclusion criteria based on trial quality.^7^ We found four more ongoing trials looking at vitamin D supplementation and pre-eclampsia registered on ClinicalTrials.gov. The largest one aims to recruit a total of 460 women across the intervention arms.

Genetic predisposition to higher 25-hydroxyvitamin D levels and supplementation are not equivalent.^35^ While a randomised controlled trial is done at a given time point or period, mendelian randomisation estimates the effect of a lifelong difference in levels of the exposure. Mendelian randomisation might therefore not be the best approach if the association between the genetic instruments and the exposure of interest varies over the life course, or if there is a critical period for an effect of the exposure on the outcome of interest.^8^ The pathogenesis of pre-eclampsia likely starts during early pregnancy^36^ and therefore women’s nutrient status before pregnancy or at conception and during the first trimester may be of particular relevance. However, we are unaware of any evidence to support the notion that the association between the genetic instruments and 25-hydroxyvitamin D level varies greatly over the life course. Furthermore, genetically determined changes in exposure levels are usually small and assumed to be uniform in the population, whereas interventions assessed in randomised controlled trials have greater effects. For all of these reasons, extrapolating findings from a mendelian randomisation analysis beyond the modest genetic effect might not be valid.^35^

### Policy implications

Debate continues about the recommendations for vitamin D intake in pregnancy.^37^ The US Institute of Medicine has set a recommended dietary allowance for vitamin D for pregnant and lactating women of 600 IU (15 µg) daily,^38^ while the UK National Health Service recommends that all adults (including pregnant women) consume about 400 IU (10 µg) each day,^39^ and the Health Council of the Netherlands suggests that all pregnant women take 400 IU (10 µg) of vitamin D daily. In Norway, the health directorate recommends that pregnant women take one tablespoon of cod liver oil each day, which also contains 400 IU (10 µg) of vitamin D.^40^ According to the World Health Organization, evidence recommending vitamin D supplementation for women during pregnancy to reduce adverse pregnancy outcomes is insufficient.^41^ Our findings support the current WHO position. Mendelian randomisation studies with a greater number of women with pre-eclampsia will provide additional evidence to support or negate a causal effect of vitamin D in pre-eclampsia. The next step (should such an effect be likely) would require large, well conducted trials of vitamin D supplementation. In particular, it is important to find out whether the benefit of vitamin D supplementation varies by ethnic group—most studies to date have included only individuals of European origin—and a broad range of baseline 25-hydroxyvitamin D levels.

### Conclusion

Mendelian randomisation analyses using the largest available sample yielded no strong evidence to support a causal effect of vitamin D status on gestational hypertension or pre-eclampsia. Further mendelian randomisation studies are needed with a larger number of women with pre-eclampsia, or which incorporate additional genetic instruments thereby increasing the proportion of 25-hydroxyvitamin D explained. In combination with adequately powered clinical trials, this could help finally establish whether vitamin D status has a role in pregnancy related hypertensive disorders.

**What is already known on this topic**

Observational studies find that women with lower levels of 25-hydroxyvitamin D are at greater risk of pre-eclampsia
Some trials of vitamin D supplementation in pregnancy suggest a potential benefit of supplementation but they are small, heterogeneous in timing and dose, and have substantial attrition
Therefore, it is unclear whether vitamin D is a cause of pre-eclampsia

**What this study adds**

A mendelian randomisation analysis of data from several European cohorts, showed no evidence to support a causal effect of 25-hydroxyvitamin D levels on risk of pre-eclampsia or gestational hypertension
Further mendelian randomisation studies are needed with a larger number of women with pre-eclampsia, or which incorporate additional genetic instruments thereby increasing the proportion of 25-hydroxyvitamin D explained

## Web extra

Extra material supplied by authors

**Web appendix:** Supplementary material

## Notes

Contributors: AF, WN, LCS, SE, and VWVJ conceived and designed the study. RM obtained access to data from the different cohorts. All authors contributed to the design of the study. MCM, KM, NW, and AB contributed to the data analysis. MCM drafted the manuscript. CB and AF are equal contributing senior authors. All authors had full access to all of the data (including statistical reports and tables) in the study and can take responsibility for the integrity of the data and the accuracy of the data analysis. All authors interpreted data and critically revised and approved the final manuscript. MCM is guarantor. The corresponding author attests that all listed authors meet authorship criteria and that no others meeting the criteria have been omitted.

Ethical approval: The different cohorts had approvals from respective national ethical committees for medical research. Ethical approval for the ALSPAC study was granted by the ALSPAC Law and Ethics Committee and the local research ethics committees. The data collection and analysis of MoBa were approved by the Norwegian Data Inspectorate and the regional committees for medical and health research ethics. The Generation R Study was approved by the local medical ethical committee of the Erasmus Medical Centre, Rotterdam. GOPEC was approved by the Trent Multicentre Research Ethics Committee, United Kingdom.

Data sharing: No additional data available.

Transparency: The lead author (MCM) affirms that the manuscript is an honest, accurate, and transparent account of the study being reported; that no important aspects of the study have been omitted; and that any discrepancies from the study as planned have been explained.
