# Supplementary material for: Vitamin D and risk of pregnancy related hypertensive disorders: mendelian randomisation study
Source: BMJ. 2018 Jun 21;361:k2167. doi: 10.1136/bmj.k2167 (PMC6008987; doi:10.1136/bmj.k2167)
Supplement: Supplementary file 1 — Web appendix: Supplementary material [file magm042168.ww.pdf]

WEBAPPENDIX

This appendix has been provided by the authors to give readers additional information about their work.

Supplement to:

Vitamin D and risk of pregnancy-related hypertensive disorders: a Mendelian randomization study

Web appendix

Contents

1. Online supplement methods p. 3
2. Supplementary tables
    - eTable 1 p. 7
    - eTable 2 p. 8
    - eTable 3 p. 10
    - eTable 4 p. 11
    - eTable 5 p. 12
    - eTable 6 p. 13
    - eTable 7 p. 14
    - eTable 8 p. 15
    - eTable 9 p. 16
    - eTable 10 p. 17
    - eTable 11 p. 18
    - eTable 12 p. 19
    - eTable 13 p. 20
    - eTable 14 p. 21
3. Supplementary figures
    - eFigure 1 p. 22
    - eFigure 2 p. 23
    - eFigure 3 p. 24
4. References p. 25

1. Online supplement methods

Study population

The Avon Longitudinal Study of Parents and Children

The Avon Longitudinal Study of Parents and Children (ALSPAC) recruited women with expected delivery dates between April 1991 and December 1992 living in a defined area of Avon, South West England.[1,2] The participation rate of invited pregnant women was 75.3%, resulting in a total of 14,541 participants. Written informed consent was obtained from all participants. Follow-up questionnaires were administered at 12, 18 and 32 gestational weeks, while information covering the last part of pregnancy was gathered 8 weeks after delivery. Information from antenatal visits and pregnancy outcomes were abstracted from medical records. The study website contains details of all available data through a fully searchable data dictionary (http://www.bristol.ac.uk/alspac/researchers/data-access/data-dictionary/). Ethical approval for the study was granted by the ALSPAC Law and Ethics Committee and the Local Research Ethics Committees. The illustration of the selection of the sample eligible for the current analysis is given in Supplement Figure 1.

The Generation R Study

The Generation R Study recruited pregnant women with an expected delivery date between April 2002 and January 2006 living in Rotterdam, the Netherlands.[3] Estimation of the precise number of eligible pregnant women is difficult, since there is no satisfactory registry of pregnancies. Therefore, the overall response rate was calculated at birth, as the children formed a prenatally recruited birth cohort. The participation rate was 61.5% at birth, resulting in 9,778 participants, out of which 8,879 women were enrolled during pregnancy. Written informed consent was obtained from all participants. Measurements during pregnancy were conducted in two research centres in early pregnancy (gestational age <18 weeks), mid-pregnancy (gestational age 18–25 weeks) and late pregnancy (gestational age >25 weeks). Four follow-up questionnaires were sent to mothers during pregnancy, at 12, 15, 20 and 30 gestational weeks. The study protocol was approved by the local Medical Ethical Committee of the Erasmus Medical Centre, Rotterdam. The illustration of the selection of the sample eligible for the current analysis is given in Supplement Figure 2.

The Norwegian Mother and Child Cohort Study

The Norwegian Mother and Child Cohort Study (MoBa) recruited pregnant women across Norway, between 1999 and 2009, at approximately 18 gestational weeks.[4,5] The participation rate of invited pregnant women was 41%. Mothers could participate with more than one pregnancy, resulting in more than 95,000 mothers and 114,000 children. Written informed consent was obtained from all participants. Follow-up questionnaires were administered at 18, 22 and 30 gestational weeks, and additional information covering the last part of pregnancy was obtained 6 months after delivery. Information gathered through MoBa follow-up questionnaires was linked to the Medical Birth Registry of Norway through unique national identification numbers. The study was approved by the Norwegian Data Inspectorate and the Regional Committee for Medical and Health Research Ethics of South East Norway.

Estimates from a sub-study in MoBa set up to validate the pre-eclampsia (PE) definition in the Medical Birth Registry of Norway (MBRN) was available for the two-sample MR analysis. All MoBa participants registered with PE in the MRBN and a random sample of those without PE were selected for a validation study, which has been previously described.[6] A subgroup of participants in this PE validation study were subsequently genotyped, which included women with singleton pregnancies, that were conceived spontaneously, with information on questionnaires from 18 and 30 gestational weeks, without a history of chronic hypertension before pregnancy. For the current study, we also excluded outliers for any of the first three 1000 Genomes axes of variation characterizing population stratification, based on >3 standard deviation from the mean. Therefore, 1,513 cases and 971 controls were included in the final analysis.

The UK Genetics of Pre-eclampsia study

The UK Genetics of Pre-eclampsia study (GOPEC) is a consortium of researchers from 10 universities across the UK, which recruited women affected by PE and their families between 1992 to 2009 for genetic studies of PE.[7] All participants gave written informed consent, and ethical approval was obtained by the Trent Multicentre Research Ethics Committee. White western European women with PE were recruited at the time of diagnosis. Women who developed hypertension or proteinuria before 20 gestational weeks were excluded, as were women with essential hypertension, diabetes, renal or cardiac disease, or a current multiple pregnancy. These exclusions resulted in 1,990 women with PE being taken forward to genotyping. Control data were derived from the WTCCC2 genome-wide analysis of 2,930 samples from the 1958 Birth Cohort, in addition to 2,737 samples from the National Blood Services, resulting in a total 5,667 genotyped controls.[8] Of the total 1,990 PE cases that were genotyped, 56 were removed because of genotyping quality, and 59 were removed

because of non-European ethnicity/relatedness/gender mismatch, which left a total of 1,875 PE cases for the final analysis. Furthermore, of the 5,667 controls that were genotyped, 406 were removed because of genotyping quality issues, and a further 173 were removed because of non-European ethnicity/relatedness/gender mismatch, which left a total of 5,088 controls available for the final analysis.

25-hydroxyvitamin D levels

In ALSPAC, serum samples taken as part of routine antenatal care were collected and stored initially at −20 °C and then at −80 °C, with no further freeze–thaw cycles. Serum samples could be from any stage of pregnancy, and dates of blood sampling were obtained from medical records and verified from the freezer storage data. Serum 25-hydroxyvitamin D2 (25(OH)D2) and 25-hydroxyvitamin D3 (25(OH)D3) levels were measured with high performance liquid chromatography-tandem mass spectrometry (LC-MS/MS) at a laboratory at the University of East Anglia (East Anglia, United Kingdom) meeting the performance target set by the Vitamin D External Quality Assessment Scheme (DEQAS).[9] Inter-assay coefficients of variation were less than 10% across a working range of 2.5–624 nmol/L for both 25(OH)D3 and 25(OH)D2.

In the Generation R Study, antenatal blood samples were collected in mid-pregnancy (median 20.3 weeks, range 18.5–23.3 weeks).[10] Plasma was stored at -80°C from collection until the assays were conducted. Plasma levels of 25(OH)D2 and 25(OH)D3 was quantified using isotope dilution LC-MS/MS at the Queensland Brain Institute (Brisbane, Australia) also approved by DEQAS. Assay accuracy was assessed at four concentration levels for 25(OH)D3 (48.3, 49.4, 76.4, 139.2 nmol/ L) and a single level for 25(OH)D2 (32.3 nmol/L), and was excellent at all concentration levels tested (<10% and <17%, respectively).[11]

Standardization of 25-hydroxyvitamin D measurement by season of blood sample collection

The 25(OH)D levels were standardized by the season of blood sample collection. The date of blood sample collection was available in ALSPAC, while the calendar week of blood sample collection was available in Generation R and MoBa. We used the following sine-cosine function of the date of blood sample collection to model the seasonal variation in natural logarithm transformed 25(OH)D levels in a linear regression, which has been described in detail in a previous publication from the ALSPAC cohort.[9]

$$f(t)= \alpha + \beta_h \sin(2h\pi t) + \theta_h \cos(2h\pi t)$$

Where α, β_h & θ_h are estimated regression parameters and t is the date of the blood sampling/calendar week of blood sampling. This function has been shown to adequately describe the normal seasonal variations in 25(OH)D throughout the year among individuals of European ethnicity.[12] The steps of the standardization was as follows. First, we generated the linearly predicted value of the woman's natural log transformed 25(OH)D level. We subsequently back transformed the linearly predicted value of 25(OH)D on the log scale to the raw scale by exponentiating the value and generated the residual as the difference in the measured and predicted 25(OH)D values on the raw scale. Finally, we generated the standardized value of the woman's 25(OH)D on the raw scale by adding the population geometric mean and this estimated residual.

Genotyping

In ALSPAC, DNA samples were extracted from whole blood samples taken during pregnancy. The Centre National de Génotypage (Evry, France) carried out DNA genotyping on the Illumina human660W-quad array and genotypes were called with Illumina GenomeStudio. PLINK version 1.07 (http://pngu.mgh.harvard.edu/purcell/plink)[13] was used to carry out Quality Control (QC) measures on an initial set of 10,015 subjects and 557,124 directly genotyped single nucleotide polymorphisms (SNPs). SNPs were removed if they displayed more than 5% missing, a Hardy-Weinberg equilibrium (HWE) p-value of less than 1.0×10^{-6}, or a minor allele frequency (MAF) of less than 1%. Samples/individuals were excluded if they displayed more than 5% missing values, had indeterminate X chromosome heterozygosity, extreme autosomal heterozygosity, or showed evidence of non-European ancestry. Multidimensional scaling of genome-wide identity was conducted by state pairwise distances using the four HapMap populations as a reference. Autosomal SNPs were imputed using Impute2 v2.2.2 and the 1000 genomes phase 1 version 3 reference panel including 2186 haplotypes from all populations. SNP call rates for rs10741657, rs12785878, rs2282679 and rs6013897 were 99.9%, 100%, 97.2% and 99.2%, respectively. All of these four SNPs used as instruments were imputed in ALSPAC, and they had a high imputation quality (R^2 >0.98).

In the Generation R Study, DNA was derived from whole blood samples in early pregnancy. DNA was extracted, plated and normalized from 5 ml whole blood at the Human Genotyping and Sequencing Facility of the Genetic Laboratory at the Department of Internal Medicine, Erasmus MC. Genotyping was performed at LGC Genomics (UK) using KASP genotyping. KASP genotyping assays are based on competitive allele-specific polymerase chain reaction and enable bi-allelic scoring of SNPs. Assays are deemed to be working successfully if clusters are distinct and call rates are consistently high. The data is automatically quality control

checked. The automatic control is performed at LGC Genomics using KlusterCaller which performs review of the spatial distribution of clustering groups, genotype calling and assessment of control wells. No Template Controls (NTCs) are included on each plate to enable the detection of contamination or non-specific amplification. All plated samples are included in the QC (n = 7,675 women). SNP call rates for rs10741657, rs12785878, rs2282679 and rs6013897 were 99.4%, 99.2%, 99.6% and 99.2%, respectively. All SNPs used as genetic instruments in our study were directly genotyped. Generation R has previously reported the accuracy of KASP genotyping observing a concordance rate of 99.4% and non-reference discordant rate of 1.7% with other genotyping technology.[14]

In MoBa, DNA was extracted manually from whole blood samples obtained at recruitment (around 18 gestational weeks) using the FlexiGene kit (Qiagen, Hilden, Germany). Mothers were genotyped by the UNC Mammalian Genotyping Core using the HumanCoreExome Bead Chip from Illumina (Illumina, Inc., San Diego, CA). Samples and SNPs were examined using PLINK 1.07 (http://pngu.mgh.harvard.edu/purcell/plink) for quality control. SNPs were excluded if the missing rate exceeded 5%, there was substantial deviation from HWE ($p < 1x10^{-3}$) or the MAF was <5%. Known genotype and DNA replicates were included on each plate and exhibited high genotyping quality. All subject-specific call rates were acceptable (minimum 97.2%). Sex-specific markers were inspected and relatedness and inbreeding within the cohort was assessed by identity by descent (IBD>0.125). For each pair of related mothers, we preferentially included the one with the most complete genetic data, or in the case of equivalence, randomly sampled between them. Quantile-quantile plots and calculation of genomic control lambda[15] (λ_{GC} =1.01) indicated no systematic test statistic inflation, unidentified relationships, or cryptic admixture. Outliers for any of the first three 1000 Genomes axes of variation (based on CEU, YRI, CHB, PUR, CLM, and MXL) >3 standard deviations from the mean were excluded. The post-QC dataset was imputed using PBWT [16] and pre-phased using SHAPEIT2 [17] against the 1000 Genomes Phase 3 reference panel [18]. Imputation was conducted by the Sanger Imputation Service provided by the Wellcome Trust Sanger Institute.[19] Three of the four SNPs were directly genotyped (rs6013897 was imputed).

In the GOPEC study, the Illumina 660 chip was used to genotype 1,990 pregnant women with PE. A total of 594,398 variants were called with the GenCall algorithm. Quality control analysis was conducted using PLINK (http://zzz.bwh.harvard.edu/plink/) and SMARTPCA21. Briefly, the quality control included the following subject-level exclusion criteria: individual call rate <98%, heterozygosity >3 s.d. from the mean; any of the first three HapMap (based on CEU, YRI, CHB, JPT and GIH populations) principal axes of variation >4 s.d. from the mean; and sex mismatch. Related individuals (identity by descent (IBD) > 0.1) with the lowest call rates were preferentially removed. The variant-level exclusion criteria were as follows: call rate <98%; exact Hardy–Weinberg equilibrium $P < 1 \times 10{-}6$; minor allele frequency (MAF) <1%; and non-random missingness of uncalled genotypes (plink–test-mishap) with Bonferroni-corrected $P < 0.05$. These filters left 1,882 samples and 508,748 variants. The GOPEC study obtained population controls from the National Blood Donors Cohort and the UK 1958 Birth Cohort. These samples were genotyped on the Illumina 1.2M chip and variants were called using GenCall. Strand-ambiguous markers were removed, and the standard QC procedure described above was then applied to the two control data sets. The merged control data set consisted of 5,121 samples and 860,427 variants. This control data set was merged with the case data set, resulting in 495,890 variants after quality for 1,875 cases and 5,088 controls. SNP call rates for rs10741657, rs12785878, rs2282679 and rs6013897 were 99.8%, 99.9%, 99.8% and 98.3%, respectively.

Cases and controls were imputed together with IMPUTE2 (impute_v2.3.0)22 and SHAPEIT23 using the prephasing workflow with the 1000 Genomes Project Phase 1 reference panel (December 2013) downloaded from the IMPUTE2 website. Imputation resulted in 11,553,589 biallelic variants with MAF >0.25% that were either directly genotyped or imputed with IMPUTE2 INFO score >0.6. This case-control datasets was densely imputed using IMPUTE2 (impute_v2.3.0)[20] and SHAPEIT2[21] using the pre-phasing workflow against the 1000 Genomes Phase 1 reference panel (Dec 2013) downloaded from the IMPUTE2 website. Three of the four genetic instruments used in the current study were imputed in the GOPEC study (rs2282679 was the only SNP directly genotyped) with a high quality score (R^2 >0.98). The final analysis sample consisted of 1,875 PE cases and 5,088 healthy controls. Post-imputation association analysis was carried out using SNPTEST (v2.4.1)22 with the "expected" method, including five principal components to account for an influence of population stratification and we subsequently applied a genomic control of λ_{GC}= 1.05.

Gestational hypertension and pre-eclampsia

In ALSPAC, information on pre-existing hypertension was available based on self-report through a questionnaire completed at recruitment, while six trained research midwives abstracted information on all measurements of blood pressure and proteinuria that were taken as part of routine antenatal care from the women's obstetric records. There was no between-midwife variation in mean values of the data abstracted, and error rates were consistently <1% in repeated data entry checks. Blood pressure measurements were taken in the

seated position with Korotkoff phase V cuff. The median (IQR) number of blood pressure and proteinuria measurements throughout pregnancy were 13 (11, 16) and 12 (9,14), respectively. Gestational hypertension (GH) was defined based on systolic blood pressure ≥140 mm Hg and/or diastolic blood pressure ≥90 mm Hg on two occasions after 20 weeks' gestation among previously normotensive women. PE was defined as GH along with proteinuria of ≥1+ (≥ 300 mg/dl) on urine dipstick testing on at least two occasions after 20 weeks' gestation.

In the Generation R Study, information on pre-existing hypertension was also available based on self-report through a questionnaire administered at the time of recruitment. Hypertensive disorders in pregnancy was defined by certified medical doctors comprehensively reviewing the participant's medical charts.[22] Women were classified with GH if they had a systolic blood pressure ≥140 mmHg and/or a diastolic blood pressure of ≥90 mmHg first occurring after 20 weeks of gestation at two time-points. These criteria and the presence of proteinuria (defined as 2 or more dipstick readings of 2 or greater, 1 catheter sample reading of 1 or greater, or a 24-hour urine collection containing ≥ 0.at least 300 mg of protein) were used to identify women with PE.

The definition of PE in the MoBa PE validation study using antenatal medical records (used for MoBa3) [6] was defined as new-onset hypertension and proteinuria after 20 gestational weeks, with systolic blood pressure ≥ 140 mmHg and/or diastolic blood pressure of ≥ 90 mmHg on at least two occasions, and proteinuria defined by urine protein ≥ 0.3 g/24-hour or 1+ on urine dipstick.

In the GOPEC study, the information used to define PE was exclusively from antenatal medical records. PE was defined based on new-onset hypertension after 20 gestational weeks, with systolic blood pressure ≥140 mmHg and/or diastolic blood pressure rose to ≥90 mmHg on two occasions, in combination with proteinuria as defined based on ≥0.3 g/24-hour or ≥1+ on dipstick testing of urine.

Dealing with missing data in multivariable analyses

In ALSPAC, missing covariable data were imputed using multivariable multiple imputation by chained equations, by generating 20 datasets with missing data imputed from a distribution of predicted missing values obtained by including 25(OH)D, GH/PE and all covariates, together with information on the four genetic instruments, in prediction models using chained equations. This method allows you to specify the type of regression that is to be used for each of the covariates. Association estimated were then obtained by summing across these datasets using Rubins rules.[23] Missing data in Generation-R was imputed according to the Fully Conditional Specification method predictive mean matching (Generation R).[24] Both of these methods assume that the data are missing at random. Distributions of observed and imputed data were consistent with each other (Supplementary Table 1).

2. Supplementary Tables

eTable 1 Distribution of background characteristics in the Avon Longitudinal Study of Parents and Children (ALSPAC) and the Generation R Study

Characteristics	ALSPAC (n=4,066)	Generation R (n=3,323)
25-hydroxyvitamin D in nmol/L, Median (IQR)	61.8 (46.1, 81.6)	64.0 (42.6, 86.3)
Age in years, Mean(SD)	28.6 (4.6)	31.2 (4.5)
Parity, %		
0	34.2	60.1
1	31.3	30.3
2	17.8	7.9
3 or more	14.5	1.5
Missing	2.2	0.2
Pre-pregnancy BMI, %		
<18.5	4.0	3.3
18.5-24.9	71.5	63.1
25-29.9	13.9	13.5
30 or higher	4.4	5.1
Missing	6.3	15.0
Education, %		
Low (Obligatory school/vocational)	23.9	4.2
Medium (High school/Secondary school)	58.2	37.0
High (At least some higher/university education)	14.1	57.2
Missing	3.7	1.7
Smoking, %		
Never smoked	50.9	67.4
Former/Until pregnancy was known[a]	25.6	8.7
Continued during pregnancy	21.5	16.1
Missing	2.1	7.8
Calcium level in mmol/L/ Energy adjusted calcium intake in mg, Mean(SD)[b]	1.4 (0.8)	1,185 (331)
Missing, %	0.2	12.6
Vitamin D supplement, %		
No	86.0	55.5
Yes	13.1	32.0
Missing	0.8	12.5
Gestational week of blood sampling, Median(IQR)	30 (15, 33)	20 (19, 21)

BMI=body-mass index.

[a] In ALSPAC, this category included individuals who were previous smokers, while in Generation R, it included individuals who smoked early in pregnancy before realizing that they were pregnant.

[b] Calcium and energy intake was estimated using food-frequency questionnaires in Generation R, while ionized calcium was measured during pregnancy in ALSPAC.

eTable 2 Distribution of background characteristics within the observed and imputed datasets in the Avon Longitudinal Study of Parents and Children (ALSPAC) and the Generation R Study

Characteristics	ALSPAC		Generation R	
	Observed	Imputed	Observed	Imputed
25-hydroxyvitamin D in nmol/L, Median (IQR)	61.8 (46.1, 81.6)	61.8 (46.1, 81.6)	64.0 (42.6, 86.3)	64.0 (42.6, 86.3)
Age in years, Mean(SD)	28.6 (4.6)	28.6 (4.6)	31.2 (4.5)	31.2 (4.5)
Parity, n(%)				
0	1,389 (34.9)	1,424 (35.0)	1,193 (60.1)	1,997 (60.1)
1	1,274 (32.0)	1,302 (32.0)	1,008 (30.4)	1,011 (30.4)
2	725 (18.2)	739 (18.2)	263 (7.9)	263 (7.9)
3 or more	588 (14.8)	601 (14.8)	51 (1.5)	52 (1.5)
Pre-pregnancy BMI, n(%)				
<18.5	161 (4.2)	173 (4.3)	108 (3.8)	129 (3.9)
18.5-24.9	2,907 (76.3)	3093 (76.1)	2,098 (74.3)	2,449 (73.7)
25-29.9	565 (14.8)	608 (15.0)	450 (15.9)	529 (15.9)
30 or higher	178 (4.7)	192 (4.7)	168 (5.9)	216 (6.5)
Education, n(%)				
Low (Obligatory school/vocational)	972 (24.8)	1,024 (25.2)	140 (4.3)	145 (4.4)
Medium (High school/Secondary school)	2,367 (60.5)	2,454 (60.4)	1,228 (37.6)	1,256 (37.8)
High (At least some higher/university education)	575 (14.7)	588 (14.5)	1,900 (58.1)	1,922 (57.8)
Smoking, n(%)				
Never smoked	2,065 (51.9)	2,108 (51.8)	2,238 (73.1)	2,446 (73.6)
Former/Until pregnancy was known[a]	1,039 (26.1)	1,061 (26.1)	289 (9.4)	321 (9.7)
Continued during pregnancy	875 (22.0)	897 (22.1)	536 (17.5)	556 (16.7)
Calcium level in mmol/L/<br>Energy adjusted calcium intake in mg, Mean(SD)[b]	1.4 (0.8)	1.4 (0.8)	1,185 (331)	1185.0 (327)
Vitamin D supplement, n(%)				
No	3,498 (86.8)	3528 (86.8)	1,884 (63.4)	2,107 (63.4)
Yes	534 (13.2)	538 (13.2)	1,063 (36.6)	1,216 (36.6)
Gestational week of blood sampling, Median(IQR)	30 (15, 33)	30 (15, 33)	20 (19, 21)	20 (19, 21)

BMI=body-mass index.

[a] In ALSPAC, this category included individuals who were previous smokers, while in Generation R, it included individuals who smoked early in pregnancy before realizing that they were pregnant.

[b] Calcium and energy intake was estimated using food-frequency questionnaires in Generation R, while ionized calcium was measured during pregnancy in ALSPAC.

eTable 3 Distribution of 25-hydroxyvitamin D levels during pregnancy by background characteristics in the Avon Longitudinal Study of Parents and Children (ALSPAC)

Characteristics	<25 nmol/L (n=133)	25-49.9 nmol/L (n=1,154)	50-74.9 nmol/L (n=1,486)	75 nmol/L or higher (n=1,293)	p-value
Age in years, Mean(SD)	27.5 (5.3)	28.2 (4.5)	28.5 (4.7)	29.3 (4.5)	<0.001
Parity, %					<0.001
0	41.4	39.0	32.9	30.6	
1	30.8	28.4	32.8	32.3	
2	7.5	16.6	18.2	19.6	
3 or more	18.1	13.5	14.1	15.4	
Missing	2.3	2.5	2.0	2.2	
Pre-pregnancy BMI, %					<0.001
<18.5	12.8	3.8	3.4	3.8	
18.5-24.9	64.7	70.4	71.3	73.4	
25-29.9	11.3	14.0	14.1	13.8	
30 or higher	2.3	5.8	4.7	2.9	
Missing	9.0	6.1	6.4	6.0	
Education, %					0.01
Low	68.4	58.6	57.9	55.6	
Medium	16.5	24.7	23.1	26.5	
High	8.3	12.7	15.3	14.8	
Missing	6.8	4.1	3.8	3.1	
Smoking, %					<0.001
Never	40.6	47.1	52.6	53.1	
Former	20.3	25.7	24.6	27.1	
Current	36.8	25.1	21.2	17.1	
Missing	2.3	2.2	1.6	2.7	
Calcium level in nmol/L, Mean(SD)	1.4 (0.8)	1.4 (0.8)	1.4 (0.8)	1.4 (0.7)	0.78
Missing, %	0	0.3	0.3	0.2	
Vitamin D supplement, %					0.05
No	81.2	87.3	87.2	84.2	
Yes	16.5	11.8	12.3	15.0	
Missing	2.3	1.0	0.6	0.9	

BMI=body-mass index; SD=standard deviation.

eTable 4 Distribution of 25-hydroxyvitamin D level during pregnancy by background characteristics in the Generation R Study

Characteristics	<25 nmol/L (n=242)	25-49.9 nmol/L (n=856)	50-74.9 nmol/L (n=993)	75 nmol/L or higher (n=1,232)	p-value
Age in years, Mean(SD)	30.2 (5.5)	30.9 (4.6)	31.4 (4.3)	31.4 (4.2)	<0.001
Parity, %					0.001
0	55.4	54.9	61.0	63.6	
1	31.0	32.4	30.4	28.7	
2	10.3	10.9	7.0	6.1	
3 or more	2.5	1.6	1.3	1.5	
Missing	0.8	0.2	0.2	0.2	
Pre-pregnancy BMI, %					<0.001
<18.5	3.3	3.6	2.7	3.4	
18.5-24.9	60.4	58.4	63.5	66.7	
25-29.9	12.4	16.6	13.3	11.9	
30 or higher	10.3	5.3	6.3	2.8	
Missing	13.6	16.1	14.2	15.2	
Education, %					<0.001
Low	9.5	5.0	3.4	3.2	
Medium	45.5	38.2	37.1	34.3	
High	42.1	54.6	58.2	61.1	
Missing	2.9	2.2	1.3	1.3	
Smoking, %					<0.001
Never smoked	56.6	65.8	68.1	70.0	
Until pregnancy was known	6.6	8.1	8.7	9.6	
Continued during pregnancy	26.0	17.9	15.7	13.3	
Missing	10.8	8.2	7.5	7.1	
Energy adjusted calcium intake in mg, Mean(SD)	1,102 (333)	1,178 (326)	1,202 (337)	1,191 (326)	0.003
Missing, %	17.8	13.3	10.4	13.0	
Vitamin D supplement, %					<0.001
No	70.7	63.0	53.3	49.1	
Yes	15.7	23.6	34.4	39.0	
Missing	13.6	13.4	12.3	11.9	

BMI=body-mass index; SD=standard deviation.

eTable 5 Distribution of background characteristics by genetic instruments in the Avon Longitudinal Study of Parents and Children (ALSPAC)

Characteristics	rs10741657				rs12785878				rs2282679				rs6013897			
	A/A (n=624)	A/G (n=1,966)	G/G (n= 1,472)	p-value	T/T (n=2,464)	T/G (n=1,371)	G/G (n= 231)	p-value	T/T (n= 1,951)	T/G (n=1,657)	G/G (n=346)	p-value	T/T (n=2,629)	A/T (n=1,267)	A/A (n=135)	p-value
Age in years, Mean(SD)	28.7 (4.6)	28.7 (4.6)	28.6 (4.7)	0.683	28.6 (4.7)	28.7 (4.4)	28.8 (4.9)	0.911	28.6 (4.6)	28.7 (4.7)	28.4 (4.6)	0.562	28.7 (4.6)	28.6 (4.7)	28.6 (4.6)	0.946
Parity, %				0.227				0.229				0.621				0.852
0	36.5	34.0	33.3		35.0	33.3	30.3		33.7	34.8	34.1		35.5	33.6	34.1	
1	32.1	31.1	31.4		30.2	32.8	35.1		32.0	29.9	34.4		31.7	30.9	29.6	
2	18.3	18.0	17.5		18.3	16.7	19.1		17.1	18.8	16.5		17.3	18.9	17.0	
3 or more	12.3	14.3	15.6		14.3	14.6	15.2		14.9	14.3	13.6		14.3	14.2	17.8	
Missing	0.8	2.6	2.3		2.2	2.6	0.4		2.3	2.1	1.5		2.2	2.4	1.5	
Pre-pregnancy, %				0.007				0.031				0.639				0.151
<18.5	4.2	3.6	4.4		3.9	4.3	2.2		3.4	4.9	2.9		3.7	4.2	6.7	
18.5-24.9	72.8	71.6	70.9		71.0	71.0	79.2		71.9	70.7	73.4		71.5	71.0	79.3	
25-29.9	14.6	14.1	13.4		14.7	13.5	7.4		13.8	14.3	12.1		13.9	14.4	7.4	
30 or higher	3.9	4.5	4.3		4.4	4.1	6.1		4.4	4.1	5.5		4.7	4.0	0.7	
Missing	4.7	6.2	7.1		5.9	7.1	5.2		6.5	6.0	6.1		6.2	6.4	5.9	
Education, %				0.957				0.150				0.455				0.615
Low	23.9	23.3	24.8		22.7	26.2	22.9		24.6	22.8	23.4		23.5	25.2	20.7	
Medium	58.5	58.7	57.5		59.5	56.3	55.4		57.5	58.5	61.3		58.7	56.9	58.5	
High	14.4	14.0	14.2		14.0	13.6	18.2		14.4	14.9	11.0		14.3	14.1	14.8	
Missing	3.2	4.1	3.5		3.7	3.9	3.5		3.5	3.8	4.3		3.6	3.9	5.9	
Smoking, %				0.456				0.016				0.087				0.838
Never	49.5	51.6	50.2		50.2	52.8	44.6		50.4	50.7	56.1		50.8	50.8	50.4	
Former	26.1	24.9	26.2		25.4	25.0	30.7		26.8	24.2	24.6		25.8	24.6	27.4	
Current	23.4	21.1	21.3		22.3	19.6	24.7		20.8	22.8	18.2		21.3	22.3	20.7	
Missing	1.0	2.4	2.2		2.1	2.6	0		2.1	2.3	1.2		2.2	2.2	1.5	
Calcium level in nmol/L, Mean(SD)	1.4 (0.8)	1.4 (0.8)	1.4 (0.8)	0.232	1.4 (0.8)	1.4 (0.8)	1.4 (0.7)	0.841	1.4 (0.8)	1.4 (0.8)	1.4 (0.7)	0.743	1.4 (0.8)	1.4 (0.8)	1.3 (0.7)	0.119
Missing, %	0.2	0.3	0.2		0.2	0.1	0.4		0.1	0.4	0.3		0.1	0.3	1.5	
Vitamin D supplement, %				0.242				0.154				0.569				0.755
No	86.7	85.9	86.0		86.3	84.9	89.6		86.7	85.3	85.8		85.8	86.7	84.4	
Yes	13.1	13.0	13.2		13.0	13.9	10.4		12.5	13.9	13.9		13.3	12.7	14.1	
Missing	0.2	1.1	0.8		0.7	1.2	0		0.8	0.8	0.3		0.9	0.6	1.5	

BMI=body-mass index; SD=standard deviation.

eTable 6 Distribution of background characteristics by genetic instruments in the Generation R Study

Characteristics	rs10741657				rs12785878				rs2282679				rs6013897			
	A/A (n=490)	A/G (n=1,580)	G/G (n= 1,233)	p-value	T/T (n=1,716)	T/G (n=1,334)	G/G (n= 244)	p-value	T/T (n= 1,714)	T/G (n=1,363)	G/G (n=232)	p-value	T/T (n=2,125)	A/T (n=1,035)	A/A (n=137)	p-value
Age in years, Mean(SD)	31.0 (4.4)	31.2 (4.4)	31.3 (4.6)	0.376	31.4 (4.4)	31.1 (4.5)	30.4 (4.4)	0.002	31.1 (4.5)	31.3 (4.5)	31.4 (4.3)	0.341	31.2 (4.4)	31.1 (4.6)	30.8 (4.6)	0.552
Parity, %				0.410				0.951				0.260				0.823
0	61.5	60.6	58.6		60.1	59.6	60.2		60.6	59.6	59.5		59.4	60.7	65.7	
1	30.0	29.7	31.4		30.2	30.4	31.1		29.4	31.3	30.2		30.6	30.1	25.5	
2	6.7	7.5	8.8		8.0	8.2	6.1		7.6	7.9	9.5		8.2	7.3	7.3	
3 or more	1.4	1.9	1.1		1.5	1.5	2.0		2.0	1.0	0.9		1.5	1.6	1.5	
Missing	0.4	0.3	0.1		0.2	0.3	0.6		0.4	0.2	0		0.3	0.3	0	
Pre-pregnancy BMI, %				0.801				0.964				0.953				0.983
<18.5	3.5	3.2	3.2		3.3	3.1	3.7		3.3	3.4	2.6		3.2	3.2	4.4	
18.5-24.9	64.7	62.7	63.1		64.1	61.7	63.9		63.7	62.8	60.8		63.2	63.1	61.3	
25-29.9	13.3	13.9	13.3		13.7	13.7	12.3		13.6	13.4	13.8		13.3	13.8	14.6	
30 or higher	4.0	5.7	4.6		5.4	4.6	5.7		5.3	4.5	6.0		5.2	4.9	4.4	
Missing	14.5	14.6	15.7		13.5	16.9	14.4		14.1	15.9	16.8		15.1	15.0	15.3	
Education, %				0.867				0.187				0.370				0.248
Low	3.7	4.3	4.2		3.6	5.0	3.7		3.6	5.1	3.9		4.0	4.7	4.4	
Medium	38.0	37.3	36.0		36.9	36.1	40.6		37.6	36.1	36.6		38.3	34.4	34.3	
High	55.3	57.1	58.2		58.4	56.7	52.5		56.9	57.7	56.5		56.2	59.0	60.6	
Missing	3.0	1.3	1.6		1.1	2.2	3.2		1.9	1.1	3.0		1.5	1.9	0.7	
Smoking, %				0.080				0.135				0.346				0.354
Never smoked	72.2	67.7	65.0		68.2	66.7	66.4		66.5	68.5	68.1		68.2	65.8	69.3	
Smoked until pregnancy was known	6.3	9.4	8.8		8.0	8.8	12.3		9.2	8.5	6.5		8.3	9.2	10.9	
Continued smoking in pregnancy	14.3	15.9	17.2		16.7	15.9	12.7		17.0	15.4	12.9		16.2	16.4	10.9	
Missing	7.2	7.0	9.0		7.1	8.6	8.6		7.3	7.6	12.5		7.3	8.6	8.9	
Energy adjusted calcium intake in mg, Mean (SD)	1,188 (353)	1,183 (322)	1,188 (325)	0.821	1,177 (324)	1,195 (332)	1,176 (327)	0.249	1,192 (320)	1,179 (340)	1,177 (287)	0.426	1,179 (332)	1,202 (334)	1,155 (289)	0.055
Missing, %	11.4	12.6	13.2		12.2	12.7	14.8		13.2	12.0	12.5		12.7	12.9	10.9	
Vitamin D supplement, %				0.650				0.166				0.979				0.656
No	58.0	54.7	55.5		56.8	53.4	57.8		55.9	55.2	53.0		55.2	55.7	54.7	
Yes	31.0	32.5	31.7		31.8	33.3	27.5		32.1	32.2	30.6		32.8	30.6	32.1	
Missing	11.0	12.8	12.8		11.4	13.3	14.7		12.0	12.6	16.4		12.0	13.6	13.2	

BMI=body-mass index; SD=standard deviation.

eTable 7 Traditional observational associations of 25-hydroxyvitamin D levels with gestational hypertension and pre-eclampsia in the Avon Longitudinal Study of Parents and Children (ALSPAC) and the Generation R Study

Study	25(OH)D (nmol/L)	N	Gestational hypertension			Pre-eclampsia		
			n	Unadjusted RR (95% CI)	Adjusted RR (95% CI)[a]	n	Unadjusted RR (95% CI)	Adjusted RR (95% CI)[a]
ALSPAC	Continuous[b]	4,066	592	1.01 (0.99 to 1.04)	1.01 (0.99 to 1.03)	77	1.05 (1.00 to 1.11)	1.04 (0.99 to 1.09)
	Categorical							
	<25	133	17	0.93 (0.55 to 1.59)	0.98 (0.56 to 1.70)	5	2.90 (1.05 to 8.02)	2.46 (0.86 to 6.99)
	25-49.9	1,154	185	1.19 (0.95 to 1.49)	1.08 (0.86 to 1.36)	23	1.57 (0.83 to 2.95)	1.28 (0.67 to 2.44)
	50-74.9	1,486	210	1.03 (0.83 to 1.27)	0.97 (0.78 to 1.21)	32	1.66 (0.92 to 3.00)	1.54 (0.84 to 2.81)
	75 or higher	1,293	180	1	1	17	1	1
Generation R	Continuous[b]	3,323	159	1.02 (0.99 to 1.05)	1.01 (0.98 to 1.05)	58	1.02 (0.97 to 1.07)	1.02 (0.97 to 1.08)
	Categorical							
	<25	242	16	1.57 (0.88 to 2.79)	1.37 (0.74 to 2.52)	7	1.85 (0.77 to 4.43)	1.77 (0.71 to 4.43)
	25-49.9	856	39	1.04 (0.68 to 1.58)	0.98 (0.63 to 1.51)	12	0.86 (0.42 to 1.78)	0.92 (0.44 to 1.93)
	50-74.9	993	50	1.16 (0.78 to 1.72)	1.00 (0.66 to 1.50)	19	1.19 (0.63 to 2.24)	1.05 (0.55 to 2.02)
	75 or higher	1,232	54	1	1	20	1	1
Random effects meta-analysis	Continuous[b]	7,389	751	1.02 (1.00 to 1.03)	1.01 (0.99 to 1.03)	135	1.04 (1.00 to 1.07)	1.03 (1.00 to 1.07)
	Categorical							
	<25	375	33	1.19 (0.72 to 1.99)	1.14 (0.76 to 1.72)	12	2.24 (1.15 to 4.35)	2.04 (1.02 to 4.07)
	25-49.9	2,010	224	1.16 (0.95 to 1.41)	1.04 (0.85 to 1.28)	35	1.19 (0.66 to 2.15)	1.11 (0.68 to 1.81)
	50-74.9	2,479	260	1.06 (0.88 to 1.28)	0.97 (0.80 to 1.18)	51	1.42 (0.92 to 2.19)	1.29 (0.83 to 2.01)
	75 or higher	2,525	234	1	1	37	1	1

25(OH)D=25-hydroxyvitamin D; RR=relative risk ; CI=confidence interval.

Measures of association obtained from multinomial logistic regression analysis.

[a]Adjusted for age, parity, pre-pregnancy BMI, education, smoking, calcium level/calcium intake and gestational week of blood sampling.

[b] Associations reflect the change in risk of the outcome per 10% decrease in 25-hydroxyvitamin D.

Approximately 15% of observations have missing information on one or more covariates in the multivariable analyses. Multiple imputation of missing covariate information was therefore conducted using chained equations, where a total of 20 imputed datasets were generated.

eTable 8 Association between the genetic instruments and 25-hydroxyvitamin D levels in the Avon Longitudinal Study of Parents and Children (ALSPAC) and the Generation R Study

Study	Locus	Chromosome	25(OH)D associated SNP	25(OH)D decreasing allele	N	Risk allele frequency / Median(IQR)	Test for HWE (p-value)	Effect on 25(OH)D (percent change per risk allele)			
								β (95% CI)	p-value	F-statistic	R^2
ALSPAC	*CYP2R1*	11	rs10741657	G	4,062	0.60	0.44	-0.70 (-2.73 to 1.31)	0.49	0.47	0.0001
	DHCR7	11	rs12785878	G	4,066	0.23	0.03	-4.10 (-6.61 to -1.85)	<0.001	12.47	0.0028
	GC	4	rs2282679	G	3,954	0.30	0.85	-7.64 (-9.97 to -5.35)	<0.001	45.14	0.0113
	CYP24A1	20	rs6013897	A	4,031	0.19	0.26	-2.48 (-5.07 to 0.06)	0.06	3.66	0.0009
	Synthesis score (rs10741657 and rs12785878)				4,062	1.82 (0.91, 2.00)	NA	-2.34 (-3.89 to -0.80)	0.01	9.03	0.0022
	Metabolism score (rs2282679 and rs6013897)				3,920	1.64 **(**0.00, 1.64)	NA	-4.69 (-6.04 to -3.34)	<0.001	48.67	0.0123
	Total score				3,916	2.70 (1.46, 3.57)	NA	-3.67 (-4.63 to -2.70)	<0.001	56.82	0.0143
Generation R	*CYP2R1*	11	rs10741657	G	3,303	0.61	0.69	-3.77 (-6.46 to -1.14)	0.005	7.95	0.0024
	DHCR7	11	rs12785878	G	3,294	0.28	0.51	-5.64 (-8.65 to -2.72)	<0.001	14.65	0.0044
	GC	4	rs2282679	G	3,309	0.28	0.08	-10.87 (-14.04 to -7.80)	<0.001	51.68	0.0154
	CYP24A1	20	rs6013897	A	3,297	0.20	0.44	-1.32 (-4.52 to 1.83)	0.411	0.676	0.0002
	Synthesis score (rs10741657 and rs12785878)				3,275	1.83 (0.92, 2.00)	NA	-4.48 (-6.45 to -2.54)	<0.001	21.02	0.0064
	Metabolism score (rs2282679 and rs6013897)				3,284	0.72 (0.00, 1.64)	NA	-6.35 (-8.16 to -4.58)	<0.001	51.60	0.0155
	Total score				3,239	2.46 (1.46, 3.57)	NA	-5.42 (-6.71 to -4.15)	<0.001	73.62	0.0222

25(OH)D=25-hydroxyvitamin D; HWE=Hardy-Weinberg equilibrium; CI=confidence interval.

eTable 9 Associations of the four genetic instruments for 25-hydroxyvitamin D with gestational hypertension and pre-eclampsia from the one-sample Mendelian randomization analysis of the Avon Longitudinal Study of Parents and Children (ALSPAC) and the Generation R Study

Genetic instrument	Study	Gestational hypertension		Pre-eclampsia	
		RR (95% CI)	I^2 p-value	RR (95% CI)	I^2 p-value
rs10741657	ALSPAC	0.91 (0.80 to 1.03)		0.97 (0.70 to 1.35)	
	Generation R	1.13 (0.89 to 1.43)		1.09 (0.74 to 1.61)	
	Random-effects meta-analysis	0.99 (0.81 to 1.22)	0.11	1.02 (0.79 to 1.31)	0.65
rs12785878	ALSPAC	0.97 (0.83 to 1.12)		1.03 (0.71 to 1.50)	
	Generation R	0.87 (0.67 to 1.13)		1.13 (0.75 to 1.69)	
	Random-effects meta-analysis	0.94 (0.83 to 1.08)	0.48	1.07 (0.82 to 1.42)	0.74
rs2282679	ALSPAC	0.96 (0.84 to 1.11)		1.09 (0.76 to 1.54)	
	Generation R	0.90 (0.69 to 1.17)		1.24 (0.83 to 1.85)	
	Random-effects meta-analysis	0.95 (0.84 to 1.07)	0.67	1.15 (0.89 to 1.50)	0.64
rs6013897	ALSPAC	0.96 (0.81 to 1.12)		0.79 (0.50 to 1.23)	
	Generation R	0.89 (0.66 to 1.19)		0.94 (0.59 to 1.50)	
	Random-effects meta-analysis	0.94 (0.82 to 1.09)	0.66	0.86 (0.62 to 1.19)	0.60

RR=relative risk; CI=confidence interval.
Measures of association obtained from multinomial logistic regression analysis. Associations reflect the additive risk of each additional copy of the risk allele associated with decreased 25-hydroxyvitamin D, and are adjusted for seven principal components to account for population stratification (ALSPAC only).

eTable 10 Causal associations of 25-hydroxyvitamin D with gestational hypertension and pre-eclampsia for each genetic instrument in a one-sample Mendelian Randomization analysis of the Avon Longitudinal Study of Parents and Children (ALSPAC) and the Generation R Study

Genetic instrument	Study	Gestational hypertension		Pre-eclampsia	
		OR (95% CI)	I^2 p-value	OR (95% CI)	I^2 p-value
rs10741657	ALSPAC	0.24 (0.04 to 0.54)		0.67 (0.004 to 121.75)	
	Generation R	1.44 (0.73 to 2.84)		1.27 (0.43 to 3.71)	
	Random-effects meta-analysis	0.72 (0.13 to 3.99)	0.071	1.24 (0.43 to 3.55)	0.81
rs12785878	ALSPAC	0.92 (0.63 to 1.35)		1.09 (0.37 to 3.20)	
	Generation R	0.77 (0.46 to 1.30)		1.24 (0.56 to 2.72)	
	Random-effects meta-analysis	0.87 (0.64 to 1.18)	0.59	1.19 (0.63 to 1.24)	0.85
rs2282679	ALSPAC	0.95 (0.78 to 1.17)		1.12 (0.70 to 1.81)	
	Generation R	0.89 (0.70 to 1.15)		1.25 (0.85 to 1.84)	
	Random-effects meta-analysis	0.93 (0.79 to 1.08)	0.69	1.20 (0.89 to 1.61)	0.73
rs6013897	ALSPAC	0.81 (0.39 to 1.67)		0.36 (0.04 to 3.13)	
	Generation R	0.38 (0.03 to 4.35)		0.61 (0.02 to 18.97)	
	Random-effects meta-analysis	0.76 (0.38 to 1.53)	0.57	0.42 (0.07 to 2.64)	0.80

OR=odds ratio; CI=confidence interval.

The causal association was estimated using instrumental variable probit regression, and associations reflect the change in risk per 10% decrease in 25-hydroxyvitamin D.

The associations are adjusted for gestational week of blood sampling and seven principal components to account for population stratification (ALSPAC only).

eTable 11 The allele frequencies of the 25-hydroxyvitamin D decreasing (risk) allele in the Norwegian Mother and Child Cohort Study (MoBa) and the UK Genetics of Pre-eclampsia Study (GOPEC)

Study	Locus	Chromosome	25(OH)D associated SNP	HWE p-value	25(OH)D decreasing allele	Risk allele frequency
MoBa (n=971 controls)	*CYP2R1*	11	rs10741657	0.51	G	0.59
	DHCR7	11	rs12785878	0.03	G	0.35
	GC	4	rs2282679	0.18	G	0.26
	CYP24A1	20	rs6013897	0.51	A	0.24
GOPEC (n=5,088 controls)	*CYP2R1*	11	rs10741657	0.93	G	0.60
	DHCR7	11	rs12785878	0.25	G	0.22
	GC	4	rs2282679	0.21	G	0.30
	CYP24A1	20	rs6013897	0.59	A	0.20

25(OH)D=25-hydroxyvitamin D; HWE=Hardy-Weinberg equilibrium.

eTable 12 Associations of the four genetic instruments for 25-hydroxyvitamin D with pre-eclampsia from the two-sample Mendelian Randomization analysis of the Norwegian Mother and Child Cohort Study (MoBa) and the UK Genetics of Pre-eclampsia Study (GOPEC)

Genetic instrument	Study	Gestational hypertension	
		OR (95% CI)	I^2 p-value
rs10741657	GOPEC	1.01 (0.94 to 1.09)	
	MoBa	1.01 (0.89 to 1.13)	
	Random-effects meta-analysis	1.01 (0.95 to 1.08)	0.95
rs12785878	GOPEC	0.95 (0.86 to 1.05)	
	MoBa	0.96 (0.85 to 1.08)	
	Random-effects meta-analysis	0.96 (0.89 to 1.03)	0.87
rs2282679	GOPEC	0.93 (0.85 to 1.02)	
	MoBa	1.06 (0.93 to 1.19)	
	Random-effects meta-analysis	0.99 (0.87 to 1.12)	0.10
rs6013897	GOPEC	1.06 (0.96 to 1.15)	
	MoBa	0.99 (0.86 to 1.12)	
	Random-effects meta-analysis	1.04 (0.96 to 1.12)	0.43

OR=odds ratio; CI=confidence interval.

The associations were estimated from ordinary logistic regression, and reflect the additive risk of each additional copy of the risk allele associated with decreased 25-hydroxyvitamin D. The estimates are adjusted for five principal components.

eTable 13 Causal association between 25-hydroxyvitamin D and pre-eclampsia for each genetic instrument from the two-sample Mendelian Randomization of the Norwegian Mother and Child Cohort Study (MoBa) and the UK Genetics of Pre-eclampsia Study (GOPEC)

Genetic instrument	Study	Gestational hypertension	
		OR (95% CI)	I^2 p-value
rs10741657	GOPEC	1.05 (0.81 to 1.35)	
	MoBa	1.03 (0.71 to 1.49)	
	Random-effects meta-analysis	1.04 (0.85 to 1.28)	0.95
rs12785878	GOPEC	0.87 (0.68 to 1.13)	
	MoBa	0.91 (0.66 to 1.25)	
	Random-effects meta-analysis	0.89 (0.73 to 1.08)	0.87
rs2282679	GOPEC	0.92 (0.83 to 1.02)	
	MoBa	1.07 (0.92 to 1.25)	
	Random-effects meta-analysis	0.98 (0.85 to 1.14)	0.11
rs6013897	GOPEC	1.35 (0.80 to 2.27)	
	MoBa	0.94 (0.46 to 1.93)	
	Random-effects meta-analysis	1.19 (0.78 to 1.81)	0.43

OR=relative risk; CI=confidence interval.

The associations are estimated using the Wald ratio, and reflect the change in risk per 10% decrease in 25-hydroxyvitamin D.

eTable 14 The causal association between 25-hydroxyvitamin D cut-off levels and pre-eclampsia for each genetic instrument from a two-sample Mendelian Randomization of the Norwegian Mother and Child Cohort Study (MoBa) and the UK Genetics of Pre-eclampsia Study (GOPEC)

Genetic instrument	Study	<75 nmol/L compared to ≥75 nmol/L		<50 nmol/L compared to ≥50 nmol/L	
		OR (95% CI)	I^2 p-value	OR (95% CI)	I^2 p-value
rs10741657	GOPEC	1.08 (0.71 to 1.63)		1.28 (0.33 to 4.96)	
	MoBa	1.05 (0.58 to 1.92)		1.18 (0.16 to 8.51)	
	Random-effects meta-analysis	1.07 (0.76 to 1.51)	0.95	1.25 (0.41 to 3.81)	0.95
rs12785878	GOPEC	0.77 (0.47 to 1.26)		0.77 (0.47 to 1.26)	
	MoBa	0.82 (0.44 to 1.53)		0.82 (0.44 to 1.53)	
	Random-effects meta-analysis	0.79 (0.54 to 1.16)	0.87	0.79 (0.54 to 1.16)	0.87
rs2282679	GOPEC	0.87 (0.73 to 1.04)		0.84 (0.68 to 1.05)	
	MoBa	1.13 (0.87 to 1.48)		1.16 (0.84 to 1.61)	
	Random-effects meta-analysis	0.97 (0.75 to 1.26)	0.11	0.97 (0.71 to 1.32)	0.11

OR=relative risk; CI=confidence interval.

The associations are estimated using the Wald ratio.

3. Supplementary figures

eFigure 1 The Avon Longitudinal Study of Parents and Children (ALSPAC)

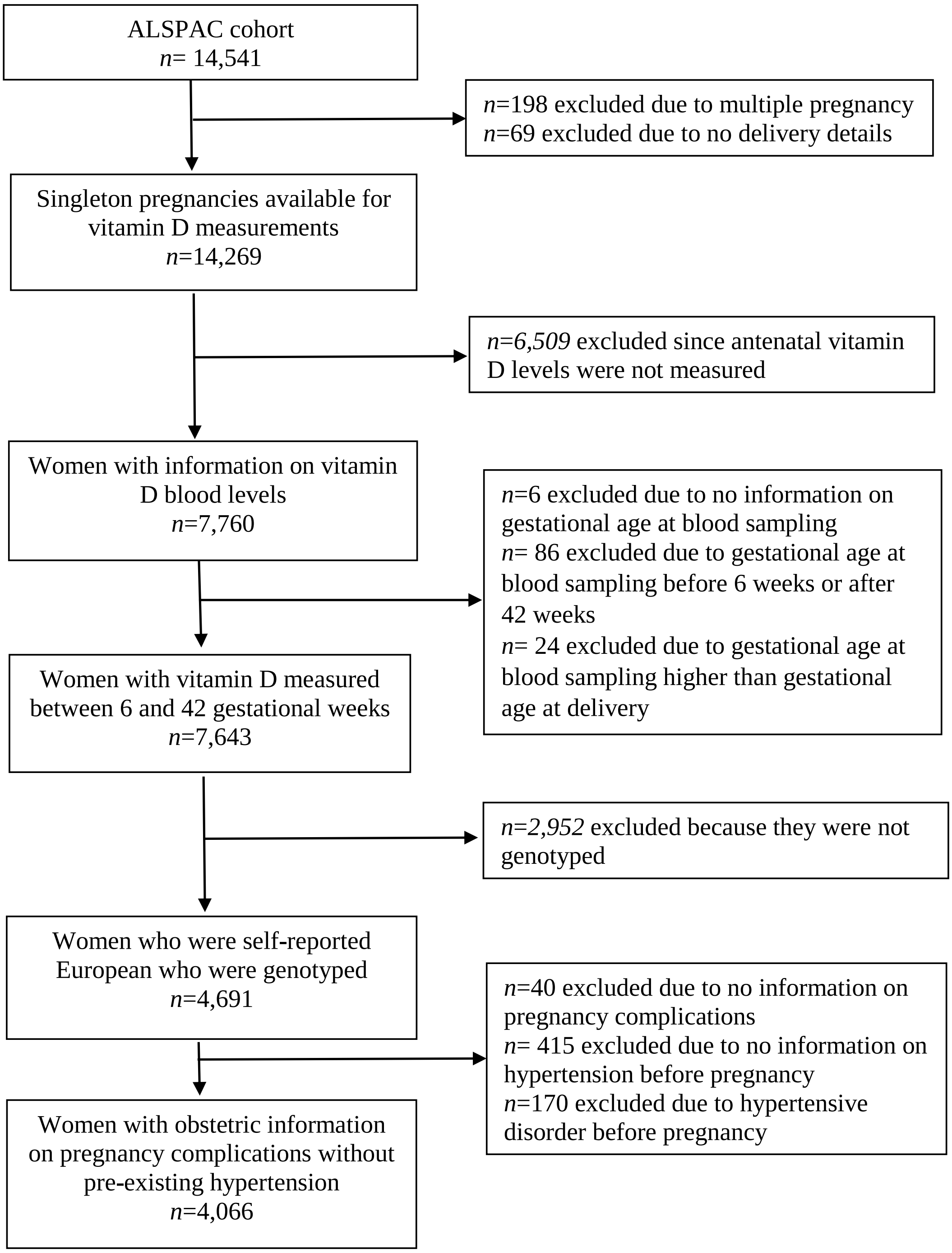

eFigure 2 The Generation R Study

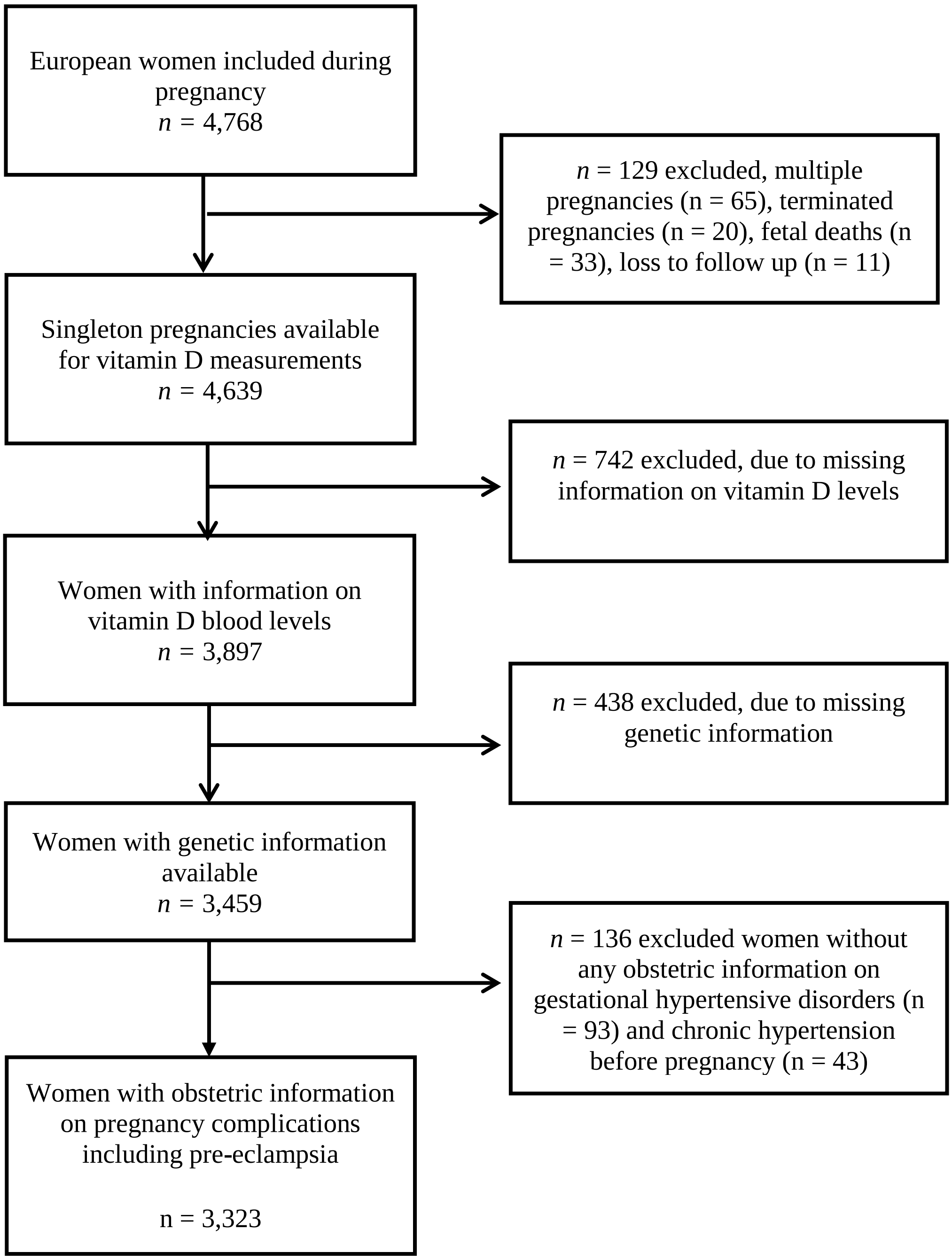

eFigure 3 Theoretical framework underlying the Mendelian Randomization analysis of vitamin D status in relation to gestational hypertension and pre-eclampsia

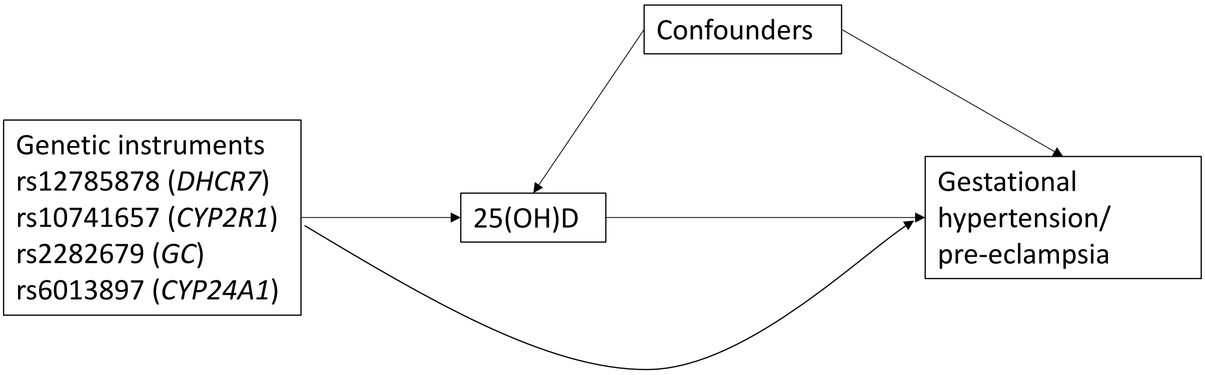

4. References

1. Fraser A, Macdonald-Wallis C, Tilling K, et al. Cohort Profile: the Avon Longitudinal Study of Parents and Children: ALSPAC mothers cohort. *Int J Epidemiol* 2013;42:97-110. doi:10.1093/ije/dys066.
2. Boyd A, Golding J, Macleod J, et al. Cohort Profile: the 'children of the 90s'--the index offspring of the Avon Longitudinal Study of Parents and Children. *Int J Epidemiol* 2013;42:111-27. doi:10.1093/ije/dys064.
3. Kooijman MN, Kruithof CJ, van Duijn CM, et al. The Generation R Study: design and cohort update 2017. *Eur J Epidemiol* 2016;31:1243-64. doi:10.1007/s10654-016-0224-9.
4. Magnus P, Birke C, Vejrup K, et al. Cohort Profile Update: The Norwegian Mother and Child Cohort Study (MoBa). *Int J Epidemiol* 2016;45:382-8. doi:10.1093/ije/dyw029.
5. Magnus P, Irgens LM, Haug K, Nystad W, Skjaerven R, Stoltenberg C. Cohort profile: the Norwegian Mother and Child Cohort Study (MoBa). *Int J Epidemiol* 2006;35:1146-50. doi:10.1093/ije/dyl170.
6. Klungsoyr K, Harmon QE, Skard LB, et al. Validity of pre-eclampsia registration in the medical birth registry of norway for women participating in the norwegian mother and child cohort study, 1999-2010. *Paediatr Perinat Epidemiol* 2014;28:362-71. doi:10.1111/ppe.12138.
7. GOPEC Consortium ~~G~~. Disentangling fetal and maternal susceptibility for pre-eclampsia: a British multicenter candidate-gene study. *Am J Hum Genet* 2005;77:127-31. doi:10.1086/431245.
8. Evans DM, Spencer CC, Pointon JJ, et al. Interaction between ERAP1 and HLA-B27 in ankylosing spondylitis implicates peptide handling in the mechanism for HLA-B27 in disease susceptibility. *Nat Genet* 2011;43:761-7. doi:10.1038/ng.873.
9. Lawlor DA, Wills AK, Fraser A, Sayers A, Fraser WD, Tobias JH. Association of maternal vitamin D status during pregnancy with bone-mineral content in offspring: a prospective cohort study. *Lancet* 2013;381:2176-83. doi:10.1016/s0140-6736(12)62203-x.
10. Miliku K, Vinkhuyzen A, Blanken LM, et al. Maternal vitamin D concentrations during pregnancy, fetal growth patterns, and risks of adverse birth outcomes. *Am J Clin Nutr* 2016;103:1514-22. doi:10.3945/ajcn.115.123752.
11. Vinkhuyzen AA, Eyles DW, Burne TH, et al. Prevalence and predictors of vitamin D deficiency based on maternal mid-gestation and neonatal cord bloods: The Generation R Study. *J Steroid Biochem Mol Biol* 2015. doi:10.1016/j.jsbmb.2015.09.018.
12. Sachs MC, Shoben A, Levin GP, et al. Estimating mean annual 25-hydroxyvitamin D concentrations from single measurements: the Multi-Ethnic Study of Atherosclerosis. *Am J Clin Nutr* 2013;97:1243-51. doi:10.3945/ajcn.112.054502.
13. Purcell S, Neale B, Todd-Brown K, et al. PLINK: a tool set for whole-genome association and population-based linkage analyses. *Am J Hum Genet* 2007;81:559-75. doi:10.1086/519795.
14. Estrada K, Styrkarsdottir U, Evangelou E, et al. Genome-wide meta-analysis identifies 56 bone mineral density loci and reveals 14 loci associated with risk of fracture. *Nat Genet* 2012;44:491-501. doi:10.1038/ng.2249.
15. Devlin B, Roeder K. Genomic control for association studies. *Biometrics* 1999;55:997-1004.
16. Durbin R. Efficient haplotype matching and storage using the positional Burrows-Wheeler transform (PBWT). *Bioinformatics* 2014;30:1266-72. doi:10.1093/bioinformatics/btu014.
17. Delaneau O, Marchini J, Zagury JF. A linear complexity phasing method for thousands of genomes. *Nat Methods* 2011;9:179-81. doi:10.1038/nmeth.1785.
18. Auton A, Brooks LD, Durbin RM, et al. A global reference for human genetic variation. *Nature* 2015;526:68-74. doi:10.1038/nature15393.
19. McCarthy S, Das S, Kretzschmar W, et al. A reference panel of 64,976 haplotypes for genotype imputation. *Nat Genet* 2016;48:1279-83. doi:10.1038/ng.3643.
20. Marchini J, Howie B. Genotype imputation for genome-wide association studies. *Nat Rev Genet* 2010;11:499-511. doi:10.1038/nrg2796.

21. O'Connell J, Gurdasani D, Delaneau O, et al. A general approach for haplotype phasing across the full spectrum of relatedness. *PLoS Genet* 2014;10:e1004234. doi:10.1371/journal.pgen.1004234.
22. Coolman M, de Groot CJ, Jaddoe VW, Hofman A, Raat H, Steegers EA. Medical record validation of maternally reported history of preeclampsia. *J Clin Epidemiol* 2010;63:932-7. doi:10.1016/j.jclinepi.2009.10.010.
23. White IR, Royston P, Wood AM. Multiple imputation using chained equations: Issues and guidance for practice. *Stat Med* 2011;30:377-99. doi:10.1002/sim.4067.
24. Sterne JA, White IR, Carlin JB, et al. Multiple imputation for missing data in epidemiological and clinical research: potential and pitfalls. *Bmj* 2009;338:b2393. doi:10.1136/bmj.b2393.
